# Higher local recurrence as the distinct failure pattern in early-onset rectal cancer: a tailored MRI score to guide therapy

**DOI:** 10.1038/s41698-025-01244-6

**Published:** 2025-12-24

**Authors:** Yunrui Ye, Ke Zhao, Lili Feng, Yanfen Cui, Zhenhui Li, Minning Zhao, Lifen Yan, Haitao Huang, Yulin Liu, Kaibo Ouyang, Weixiong Xu, Zaiyi Liu, Yong Li, Changhong Liang, Min-Er Zhong

**Affiliations:** 1 Guangdong Cardiovascular Institute, Guangdong Provincial People’s Hospital, Guangdong Academy of Medical Sciences, Guangzhou, China; 2 Department of Radiology, Guangdong Provincial People’s Hospital, Guangdong Academy of Medical Sciences, Southern Medical University, Guangzhou, China; 3 Guangdong Provincial Key Laboratory of Artificial Intelligence in Medical Image Analysis and Application, Guangzhou, China; 4 Medical Research Institute, Guangdong Provincial People’s Hospital, Guangdong Academy of Medical Sciences, Southern Medical University, Guangzhou, China; 5 Chinese Medicine Guangdong Laboratory, Hengqin, Guangdong China; 6 Department of Radiology, Sun Yat-sen University Cancer Center, Guangzhou, China; 7 State Key Laboratory of Oncology in South China, Collaborative Innovation Center for Cancer Medicine, Sun Yat-Sen University Cancer Center, Guangzhou, China; 8 Department of Radiology, Shanxi Cancer Hospital, Shanxi Medical University, Taiyuan, China; 9 Department of Radiology, the Third Affiliated Hospital of Kunming Medical University, Yunnan Cancer Hospital, Yunnan Cancer Center, Kunming, China; 10 Department of Radiology, Zhujiang Hospital, Southern Medical University, Guangzhou, China; 11 School of Medicine South China University of Technology, Guangzhou, China; 12 Department of Radiology, The Sixth Affiliated Hospital, Sun Yat-sen University, Guangzhou, China; 13 Department of Gastrointestinal Surgery, Department of General Surgery, Guangdong Provincial People’s Hospital, Guangdong Academy of Medical Sciences, Southern Medical University, Guangzhou, China

**Keywords:** Cancer, Oncology

## Abstract

The unique biology of early-onset locally advanced rectal cancer (EOLARC) may drive distinct failure patterns, challenging current age-agnostic management. This multicenter retrospective study aimed to define these patterns compared to late-onset disease (LOLARC) and develop a tailored magnetic resonance imaging (MRI)-based risk score. We analyzed 1289 patients undergoing neoadjuvant chemoradiotherapy and surgery. After propensity score matching, EOLARC patients exhibited higher local recurrence rates (4.9% vs. 1.7%; *P* = 0.03) despite similar pathological complete response (pCR) rates (25.5% vs. 21.2%; *P* = 0.22). The novel mrTML score (incorporating tumor deposits, mesorectal fascia, and lateral lymph nodes) effectively predicted local recurrence (adjusted hazard ratio for score 3 vs. 0, 33.99). Notably, high pre-treatment risk persisted even among patients achieving pCR (HR 12.02; *P* = 0.003), highlighting failure patterns uncaptured by pathological response. The mrTML score is a robust tool to identify patients at high risk for local recurrence, providing an evidence-based framework for risk-adapted therapy.

## Introduction

While the overall incidence of colorectal cancer has declined, a concerning and divergent trend has emerged: a steady increase in both the incidence and mortality of early-onset rectal cancer in individuals younger than 50 years^1–4^. This increase is particularly pronounced in patients presenting with locally advanced disease (early-onset locally advanced rectal cancer (EOLARC)), who constitute a growing and clinically challenging population^5,6^. These young patients often face a lifetime of consequences from aggressive multimodal therapies. These include significant bowel and sexual dysfunction and the potential for a permanent stoma, which dramatically compromises their quality of life during their most productive years^7–9^.

Accumulating evidence suggests that EOLARC is not merely “rectal cancer in the young” but a biologically distinct entity. Compared to late-onset disease (LOLARC; diagnosed age ≥50 years), it is often characterized by more aggressive clinicopathological features and unique molecular signatures, such as more advanced disease stages and poor differentiation, differences in microsatellite instability, CpG island methylation phenotype, and chromosomal instability^10–13^. Despite this recognized heterogeneity, current international practice guidelines remain age-agnostic, lacking a tailored risk stratification pathway for this population^14,15^. This “one-size-fits-all” approach creates a critical clinical dilemma: young patients are exposed to a dual jeopardy of potential undertreatment, risking recurrence, or overtreatment with aggressive regimens that offer controversial survival benefits^16–18^. Therefore, establishing a reliable, EOLARC-specific risk stratification tool to guide more precise, personalized therapy and resolve this clinical dilemma is a crucial unmet need.

Preoperative magnetic resonance imaging (MRI) is the standard for risk stratification in rectal cancer, providing crucial information on local tumor extent and key prognostic features that guide neoadjuvant therapy decisions^19–24^. However, the utility of existing MRI-based risk models in the context of EOLARC is limited. These models were developed predominantly in older or mixed-age populations^25–27^, and their accuracy in the distinct biological setting of EOLARC is questionable, representing a significant gap in current risk assessment tools. Beyond pre-treatment staging, the prognostic significance of pathological complete response (pCR)—a key therapeutic goal and the principal surrogate for long-term survival^28^—is also being challenged in this young cohort. A growing body of evidence in general rectal cancer populations suggests that a subset of patients, particularly those with high-risk features, may still experience recurrence despite achieving pCR, questioning its universal applicability^29,30^. It remains critically unclear whether pCR holds the same definitive prognostic value in EOLARC or if its significance is overshadowed by the inherent biological aggressiveness captured by pre-treatment imaging. This uncertainty represents a major barrier to achieving true personalized therapy for young patients.

**Fig. 1:**
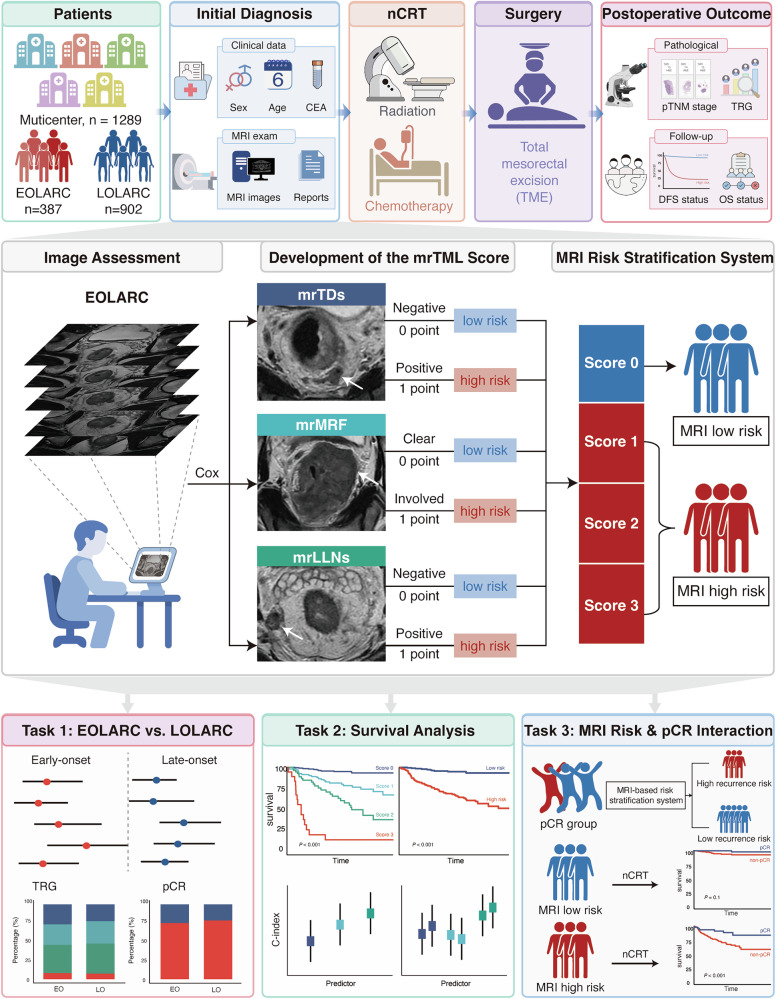
Study design. This multicenter study enrolled 1289 patients. The mrTML score was developed based on three MRI features, stratifying patients into low-risk or high-risk groups. Key findings include comparisons between propensity-matched EOLARC and LOLARC, survival analysis by mrTML score, and re-evaluation of pCR value based on MRI risk.

To address these critical gaps, this multicenter study was designed to (1) characterize the distinct patterns of treatment failure in EOLARC; (2) develop a novel, EOLARC-specific MRI-based risk score (the mrTML score); and (3) use this score to re-evaluate the prognostic role of pCR, aiming to establish a framework for personalized therapy (Fig. 1).

## Results

### Clinicopathological difference between early-onset and late-onset LARC patients

A total of 1289 patients with LARC from five institutions were included in the final analysis (Supplementary Fig. 1), comprising 387 EOLARC patients (median age 43 years [interquartile range (IQR), 37–46]; 64.3% male) and 902 LOLARC patients (median age 61 years [IQR 55–66], 67.8% male). The median follow-up for the entire cohort was 3.62 years (IQR: 2.13–4.74). Follow-up duration was similar between young-onset and late-onset patients (3.65 years [2.27–4.88] vs. 3.59 years [2.04–4.67]), with no significant difference observed (log-rank *P* = 0.10). At baseline, significant differences were observed between the two cohorts (Table 1). The EOLARC cohort presented with a more aggressive radiological profile, characterized by a higher rate of advanced mrT stage (46.8% vs. 34.6%; *P* < 0.001), tumor deposits (mrTDs, 23.3% vs. 17.4%; *P* = 0.02) and lateral lymph nodes (mrLLNs) involvement (14.7% vs. 10.3%; *P* = 0.03). Interestingly, this was accompanied by a higher clinical stage III burden (86.3% vs. 81.5%; *P* = 0.04), despite a lower prevalence of elevated preoperative CEA levels (34.1% vs. 42.4%; *P* < 0.01). Exploratory molecular analysis in a subset of patients (*N* = 274) demonstrated a trend toward higher frequencies of specific alterations in the EOLARC group, although these differences did not reach statistical significance likely due to the limited sample size (Supplementary Table 1). Specifically, microsatellite instability (MSI, 10.1% vs. 6.2%) and *KRAS* mutations (11.4% vs. 9.2%) were elevated in the early-onset group. To mitigate the imbalances, we performed propensity score matching (PSM).

**Table 1:** Baseline characteristics in patients with early-onset and late-onset locally advanced rectal cancer

Characteristics	All patients *N* = 1289	EOLARC *N* = 387	LOLARC *N* = 902	*P* value
Age (y)^a^	56 [47–63]	43 [37–46]	61 [55–66]	<0.001
Gender				0.25
Male	861 (66.8)	249 (64.3)	612 (67.8)	
Female	428 (33.2)	138 (35.7)	290 (32.2)	
CEA level^b^				<0.01
Normal	772 (60.0)	255 (65.9)	517 (57.3)	
Abnormal	514 (40.0)	132 (34.1)	382 (42.4)	
cTNM stage				0.04
II	220 (17.1)	53 (13.7)	167 (18.5)	
III	1069 (82.9)	334 (86.3)	735 (81.5)	
Location				0.14
Low	601 (46.6)	193 (49.9)	408 (45.2)	
Middle-high	688 (53.4)	194 (50.1)	494 (54.8)	
MAC				0.06
Negative	1218 (94.5)	358 (92.5)	860 (95.3)	
Positive	71 (5.5)	29 (7.5)	42 (4.7)	
mrT stage				<0.001
T2-T3a/b	796 (61.8)	206 (53.2)	590 (65.4)	
T3c/d-T4	493 (38.2)	181 (46.8)	312 (34.6)	
mrN stage				0.15
N0	437 (33.9)	143 (37.0)	294 (32.6)	
N1-N2	852 (66.1)	244 (63.0)	608 (67.2)	
mrTDs status				0.02
Negative	1042 (80.8)	297 (76.7)	745 (82.6)	
Positive	247 (19.2)	90 (23.3)	157 (17.4)	
mrEMVI status				0.22
Negative	928 (72.0)	269 (69.5)	659 (73.1)	
Positive	361 (28.0)	118 (30.5)	243 (26.9)	
mrMRF involvement				1.00
Negative	880 (68.3)	264 (68.2)	616 (68.3)	
Positive	409 (31.7)	123 (31.8)	286 (31.7)	
mrLLNs status				0.03
Negative	1139 (88.4)	330 (85.3)	809 (89.7)	
Positive	150 (11.6)	57 (14.7)	93 (10.3)	
pTNM				0.10
0/I	599 (46.5)	173 (44.7)	426 (47.2)	
II	386 (29.9)	108 (27.9)	278 (30.8)	
III	304 (23.6)	106 (27.4)	198 (22.0)	
TRG^c^				0.23
0	285 (22.1)	100 (25.8)	185 (20.5)	
1	362 (28.1)	103 (26.6)	259 (28.7)	
2	518 (40.2)	149 (38.5)	369 (40.9)	
3	107 (8.3)	32 (8.3)	75 (8.3)	
NA	17 (1.3)	3 (0.8)	14 (1.6)	
R0 resection status				1.00
R0 (negative margin)	1099 (85.3)	324 (83.7)	775 (85.9)	
R1 (positive margin)	4 (0.3)	1 (0.3)	3 (0.3)	
NA	186 (14.4)	62 (16.0)	124 (14.8)	
Surgical procedure type^d^				0.17
Sphincter-preserving surgery	797 (61.8)	225 (58.1)	572 (63.4)	
Abdominoperineal Resection	306 (23.7)	100 (25.8)	206 (22.8)	
NA	186 (14.5)	62 (16.1)	124 (13.8)	
Adjuvant chemotherapy				0.01
Yes	401 (31.1)	128 (33.1)	273 (30.3)	
No	106 (8.2)	20 (5.1)	86 (9.5)	
NA	782 (60.7)	239 (61.8)	543 (60.2)	
Local recurrence				0.01
No	1061 (82.3)	305 (78.8)	756 (83.8)	
Yes	42 (3.3)	20 (5.2)	22 (2.4)	
NA	186 (14.4)	62 (16.0)	124 (13.7)	
Distant metastasis				0.93
No	930 (72.2)	275 (71.1)	655 (72.6)	
Yes	173 (13.4)	50 (12.9)	123 (13.6)	
NA	186 (14.4)	62 (16.0)	124 (13.7)	
Total recurrence				0.77
No	1031 (80.0)	312 (80.6)	719 (79.7)	
Yes	258 (20.0)	75 (19.4)	183 (20.3)	

*P* values represent the comparison of clinicopathologic and imaging variables between the EOLARC and LOLARC groups, and *P* < 0.05 was considered statistically significant.

*EOLARC* early-onset locally advanced rectal cancer, *LOLARC* late-onset locally advanced rectal cancer, *CEA* carcinoembryonic antigen, *cTNM stage* clinical tumor-node-metastasis stage, *MAC* mucinous adenocarcinoma, *mr* magnetic resonance, *TDs* tumor deposits, *EMVI* extramural vascular invasion, *MRF* mesorectal fascia, *LLNs* lateral lymph nodes, *pTNM* pathological TNM stage, *TRG* tumor regression grade.

^a^Data are medians, with IQRs in parentheses.

^b^The normal values for CEA level range from 0 to 5 ng/ml. Three cases could not be assessed for normal CEA levels due to lack of archival material.

^c^Seventeen cases could not be assessed for TRG due to lack of archival material.

^d^Sphincter-preserving surgery includes Dixon, Parks, Intersphincteric Resection, Bacon, and TaTME.

### Distinct local recurrence pattern and prognostic landscape in EOLARC

Following one-to-one PSM, 348 patient pairs were identified with well-balanced baseline characteristics (all standardized mean difference (SMD) < 0.1, *P* > 0.05, Supplementary Table 2). In this matched cohort, while disease-free survival (DFS) and overall survival (OS) were comparable between groups (Supplementary Fig. 2), a clear divergence in failure patterns emerged. Even after controlling for staging differences, the EOLARC cohort still exhibited a significantly higher rate of local recurrence than their late-onset counterparts (4.9% vs. 1.7%; *P* = 0.03; Supplementary Table 2), whereas the rates of distant metastasis remained similar between the two groups (11.8% vs. 13.2%; *P* = 0.63). This implies that anatomical stage alone cannot explain the poorer outcomes in younger patients. To further address the potential confounding effects of surgical factors, we performed a subgroup analysis of patients with R0 resection. Results showed that even in this population, the EOLARC group maintained a significantly higher local recurrence rate than the LOLARC group (5.8% vs. 2.1%, *P* = 0.03).

**Fig. 2:**
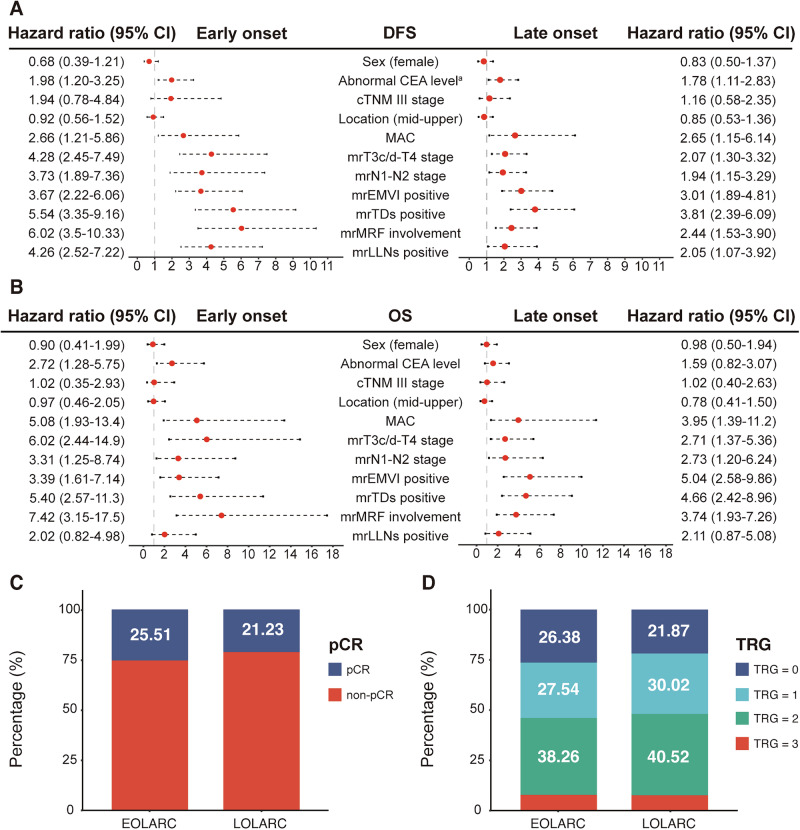
Prognostic factors and treatment response in matched EOLARC and LOLARC cohorts. **A**, **B** Forest plots of hazard ratios from univariate Cox regression for DFS and OS. **C**, **D** Distribution of TRG and pCR rates.

To explore the underlying determinants of these survival patterns, further analyses were performed within the matched cohorts. Univariate analysis revealed significant age-related disparities in the prognostic impact of MRI risk factors (Fig. 2A, B). The prognostic weight of several key features was substantially increased in younger patients. For instance, mesorectal fascia (mrMRF) involvement confers a nearly two-fold greater hazard in EOLARC for both DFS (hazard ratio [HR], 6.02 vs. 2.44) and OS (HR, 7.42 vs. 3.74). This amplified risk in younger patients also extended to mrTDs (DFS HR, 5.54 vs. 3.81) and mrLLNs (DFS HR, 4.26 vs. 2.05). Conversely, extramural vascular invasion (mrEMVI) had a more pronounced effect on OS in the LOLARC group (HR, 5.04 vs. 3.39). Particularly, this amplified risk persisted in a subgroup analysis restricted to Stage III disease, where high-risk MRI features maintained a stronger association with recurrence in the early-onset group (Supplementary Fig. 3). These findings highlight that the prognostic significance of individual risk factors is not uniform across age groups, underscoring the unique tumor biology influencing outcomes in EOLARC.

Intriguingly, the heightened local recurrence risk in EOLARC was not reflected in the pathological response to neoadjuvant chemoradiotherapy (nCRT). After matching, rates of pCR (25.5% vs. 21.2%; *P* = 0.22, Fig. 2C) and the overall distribution of tumor regression grade (TRG 0: 26.4% vs. 21.9%, *P* = 0.56, Fig. 2D) were statistically indistinguishable between the two cohorts. This reveals a critical clinical disconnect: despite achieving comparable pCR rates, younger patients still experienced a higher rate of local recurrence.

### Performance of the mrTML score

**Fig. 3:**
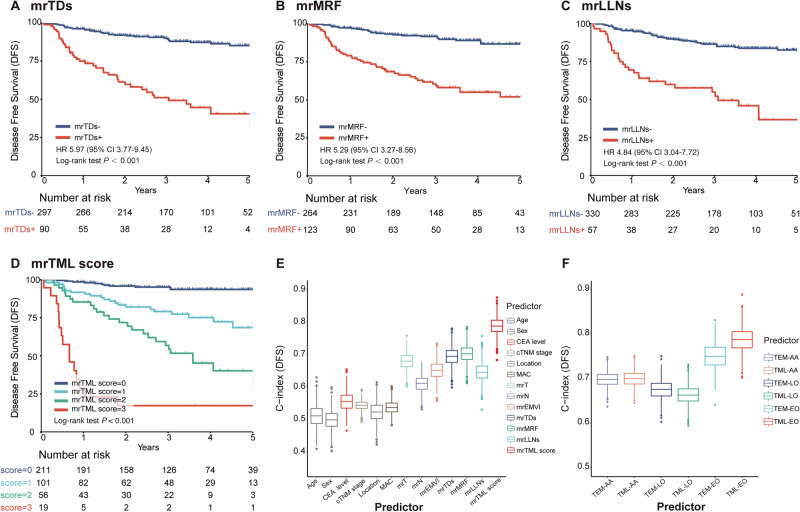
Prognostic performance of the mrTML score. **A**–**D** Kaplan–Meier curves for DFS stratified by individual MRI predictors and the composite mrTML score. **E** C-index comparison of the mrTML score vs. individual predictors. **F** C-index comparison of the mrTML vs. the mrTEM score across all-age (AA), early-onset (EO), and late-onset (LO) cohorts.

Based on three independent predictors identified via multivariable analysis (mrMRF, mrTDs, and mrLLNs; Supplementary Table 3), we developed the mrTML score (range, 0–3). Each of these individual biomarkers was significantly associated with worse survival (Fig. 3A–C, Supplementary Fig. 4A–C). Comprehensive descriptive statistics of MRI biomarkers are presented in Supplementary Table 4. This score demonstrated high interobserver reliability across radiologists with varying experience levels (Cohen’s κ, 0.76–0.84; Supplementary Table 5).

The mrTML score powerfully stratified patients into four distinct prognostic tiers with significantly different survival outcomes (Supplementary Table 6). A steep, dose-response relationship was observed between the mrTML score and survival. As the score increased from 0 to 3, the 3-year DFS rate dropped from 95.13% to 79.03%, 56.79%, and ultimately to 17.22% for each respective tier (Fig. 3D). Similaryly, the 5-year OS rate demonstrated a comparable decline, falling from 97.82% for patients with a score of 0, to 79.82%, 69.80%, and 54.20% for scores 1, 2, and 3, respectively (Supplementary Fig. 4D). Kaplan–Meier analysis confirmed that these differences were highly significant (*P* < 0.001 for both DFS and OS). After multivariable adjustment, the mrTML score remained a potent and independent predictor of both DFS and OS, with a score of 3 conferring a more than 30-fold increased hazard for DFS (Table 2). A similar stepwise increase in risk was observed for OS, with adjusted hazard ratios (AHR) of 10.44, 13.31, and 31.19 for scores 1, 2, and 3, respectively. Notably, the score also predicted the pattern of failure, with the highest score (score 3) exhibiting markedly increased risks of both local recurrence (AHR, 33.99) and distant metastasis (AHR, 20.26) (Supplementary Fig. 5 and Supplementary Table 7).

**Table 2:** Univariable and multivariate cox regression analyses in EOLARC patients for DFS and OS

	Disease-free survival				Overall survival			
	Univariate analysis		Multivariate analysis		Univariate analysis		Multivariate analysis	
	HR (95% CI)	*P*	AHR (95% CI)	*P*	HR (95% CI)	*P*	AHR (95% CI)	*P*
Age	1.00 (0.97–1.04)	0.95			0.95 (0.91–0.99)	0.02		
Gender								
Male	Reference				Reference			
Female	0.96 (0.59–1.54)	0.85			1.31 (0.67–2.54)	0.43		
CEA level^a^								
Normal	Reference				Reference		Reference	
Abnormal	1.72 (1.09–2.71)	0.02			2.72 (1.41–5.26)	<0.01	2.10 (1.08–4.09)	0.03
cTNM stage								
II	Reference				Reference			
III	2.30 (0.93–5.70)	0.07			0.98 (0.38–2.53)	0.97		
Location								
Low	Reference				Reference			
Middle and high	0.85 (0.54–1.33)	0.48			0.97 (0.50–1.86)	0.92		
MAC								
Negative	Reference				Reference		Reference	
Positive	2.37 (1.25–4.50)	0.01			3.45 (1.51–7.88)	<0.01	2.13 (0.92–4.92)	0.08
mrT stage								
T2-T3a/b	Reference		Reference		Reference			
T3c/d-T4	4.60 (2.71–7.83)	<0.001	1.59 (0.87–2.91)	0.13	5.99 (2.62–13.68)	<0.001		
mrN stage								
N0	Reference				Reference			
N1-2	3.20 (1.78–5.73)	<0.001			3.10 (1.35–7.10)	0.01		
mrEMVI status								
Negative	Reference				Reference			
Positive	3.39 (2.15–5.34)	<0.001			2.85 (1.48–5.49)	<0.01		
mrTDs status								
Negative	Reference				Reference			
Positive	5.97 (3.77–9.45)	<0.001			5.57 (2.88–10.78)	<0.001		
mrMRF status								
Negative	Reference				Reference			
Positive	5.29 (3.27–8.56)	<0.001			6.44 (3.11–13.37)	<0.001		
mrLLNs status								
Negative	Reference				Reference			
Positive	4.84 (3.04–7.72)	<0.001			2.47 (1.19–5.12)	0.02		
mrTML score								
0	Reference		Reference		Reference		Reference	
1	5.08 (2.48–10.42)	<0.001	4.17 (1.94–8.95)	<0.001	12.62 (3.67–43.36)	<0.001	10.44 (3.01–36.22)	<0.001
2	11.89 (5.86–24.12)	<0.001	9.13 (4.17–19.98)	<0.001	16.58 (4.62–59.48)	<0.001	13.31(3.67–48.29)	<0.001
3	42.48 (19.3–93.6)	<0.001	30.87 (12.8–74.7)	<0.001	36.90 (9.21–147.9)	<0.001	31.19 (7.71–126.1)	<0.001

*P* value was performed by χ2 test.

*EOLARC* early-onset locally advanced rectal cancer, *HR* hazard ratio, *AHR* adjusted hazard ratio, *CI* confidence interval, *CEA* carcinoembryonic antigen, *MAC* mucinous adenocarcinoma, *mr* magnetic resonance, *EMVI* extramural vascular invasion, *TDs* tumor deposits, *MRF* Mesorectal Fascia, *LLNs* lateral lymph nodes, *mrTML score* the composite score integrating mrTDs, mrMRF, and mrLLNs status.

^a^The normal values for CEA level range from 0 to 5 ng/ml.

The mrTML score demonstrated superior discriminative performance for predicting both DFS (C-index, 0.787) and OS (C-index, 0.779) compared to conventional predictors like cTNM stage (C-index, 0.543) or any individual MRI biomarker (Fig. 3E; Supplementary Fig. 6A and Supplementary Table 8). Furthermore, the mrTML score also outperformed a conventional MRI risk model based on mrEMVI (the mrTEM score, composite score of mrTDs, mrEMVI, and mrMRF) specifically within the EOLARC population. For DFS prediction, the C-index of the mrTML score was 0.787 (95% CI: 0.735–0.834), compared to 0.750 (95% CI: 0.694–0.807) for the mrTEM score (Fig. 3F). A similar advantage was observed for OS prediction (C-index, 0.779 vs. 0.777), although the difference was less pronounced (Supplementary Fig. 6B). While the score maintained competitive prognostic performance in the late-onset (C-index for DFS, 0.664) and all-age (C-index for DFS, 0.700) cohorts, its discriminative power was maximal in the EOLARC population for which it was developed (Fig. 3F).

### Clinical utility of MRI-based risk stratification and re-evaluation of pCR

**Fig. 4:**
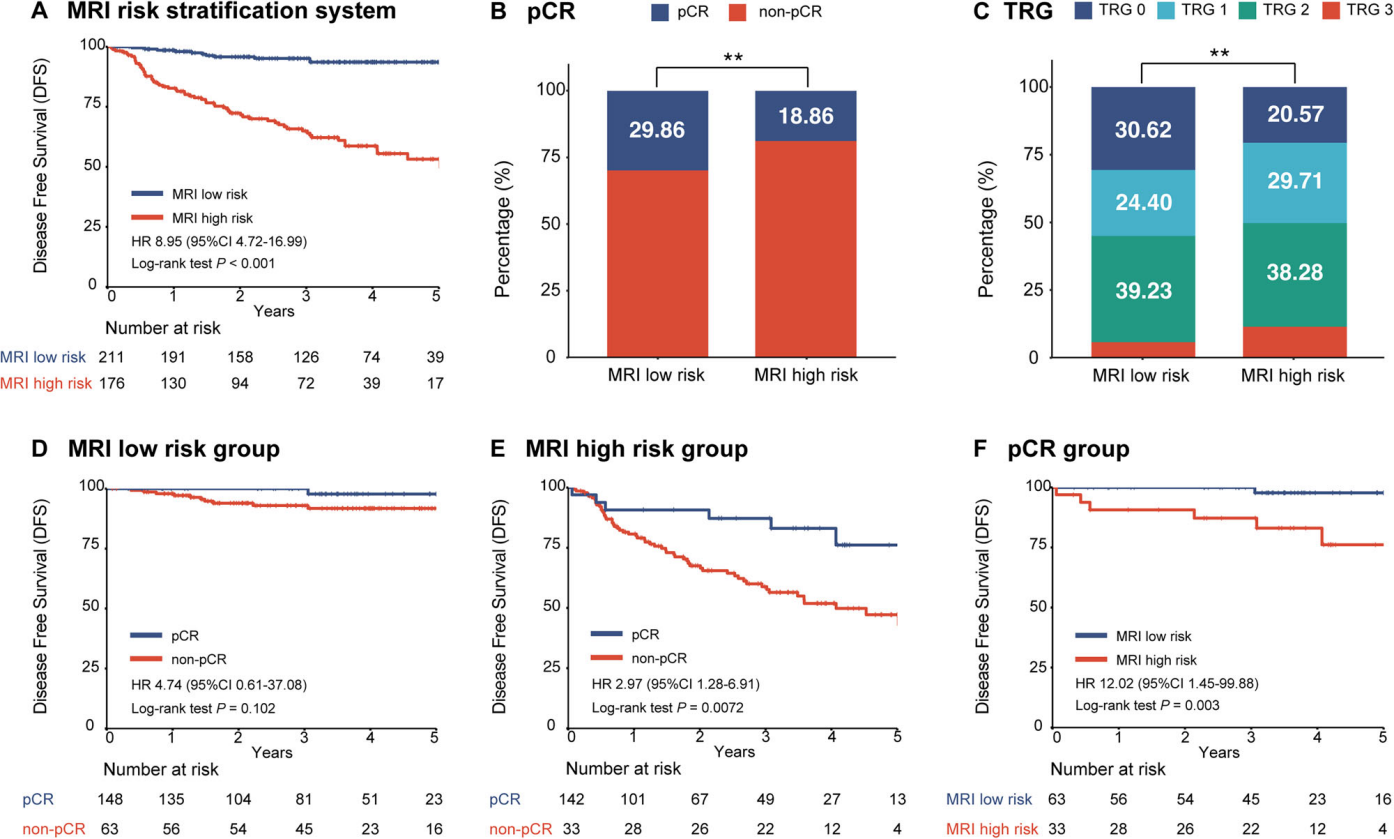
Clinical utility of the MRI-based risk stratification system in EOLARC. **A** Kaplan–Meier curves for DFS stratified by risk group. **B**, **C** Rates of pCR and TRG distributions stratified by MRI risk group. **D**, **E** Prognostic value of pCR in low- and high-risk groups. **F** Risk of recurrence among early-onset patients with pCR. Note: all hazard ratios represent the risk relative to the favorable reference group (MRI low-risk or pCR).

For clinical application, we also evaluated a simplified binary risk stratification system, defining patients with a mrTML score of ≥1 as high-risk. This system stratified 45.5% of the EOLARC cohort into a high-risk category, which faced a markedly increased hazard of both disease recurrence (HR, 8.95; 95% CI: 4.72–16.99) and mortality (HR, 15.80; 95% CI: 4.84–51.56) compared to the low-risk group (both *P* < 0.001; Fig. 4A and Supplementary Fig. 7).

This MRI-based risk stratification demonstrated a strong inverse correlation with therapeutic response. The high-risk group exhibited significantly lower rates of both pCR (18.86% vs. 29.86%; *P* = 0.017) and complete pathological response (TRG 0) compared to the low-risk group, and the likelihood of achieving pCR progressively decreased as the mrTML score increased (Fig. 4B, C and Supplementary Fig. 8). Among EOLARC patients who achieved pCR, the MRI low-risk group experienced 0.0% local recurrence, whereas the high-risk group had a recurrence rate of 3.7%, yielding an absolute difference of 3.7%. In the overall EOLARC cohort, the absolute difference in local recurrence between the MRI low-risk and high-risk groups was considerably larger at 12.2% (1.0% vs. 13.2%).

Furthermore, this pre-treatment risk stratification provided additional prognostic value that complements pathological response assessment. Within the low-risk group, achieving pCR conferred no significant survival advantage over a non-pCR outcome in either DFS or OS (both *P* ≥ 0.1; Fig. 4D and Supplementary Fig. 9A). Conversely, within the high-risk group, achieving pCR remained critical for survival, associated with significantly better outcomes in both DFS (*P* < 0.01; Fig. 4E) and OS (*P* < 0.001; Supplementary Fig. 9B). Most importantly, pre-treatment MRI risk provided further stratification even within the favorable pCR subgroup, with high-risk patients exhibiting a significantly increased hazard of recurrence (HR, 12.02; 95% CI: 1.45–99.88; *P* = 0.003, Fig. 4F).

### Adjuvant chemotherapy patterns and treatment benefit analysis

Across the entire cohort, EOLARC patients received adjuvant chemotherapy more frequently than LOLARC patients (33.1% vs. 30.3%; *P* = 0.01), and this difference persisted after PSM (32.5% vs. 29.0%; *P* = 0.03). We further analyzed the interaction between preoperative MRI-defined risk and the therapeutic benefit of adjuvant chemotherapy (Supplementary Fig. 10). Among patients receiving adjuvant chemotherapy, MRI high-risk status was significantly associated with worse disease-free survival (HR: 8.48, 95% CI: 2.57–27.97, *P* < 0.001), whereas this discrimination was attenuated in those without adjuvant therapy (HR: 2.53, 95% CI: 0.5–12.69, *P* = 0.24). Critically, treatment-stratified analyses demonstrated differential benefit across the MRI-based risk system. In the MRI low-risk subgroup, patients not receiving adjuvant chemotherapy exhibited a trend toward worse disease-free survival (HR: 5.41, 95% CI: 0.89–32.8, *P* = 0.039), although the wide confidence interval warrants cautious interpretation. In contrast, in the MRI high-risk subgroup, standard adjuvant chemotherapy failed to confer a significant survival benefit (HR: 1.92, 95% CI: 0.79–4.67, *P* = 0.14).

### Sub-analysis of very early-onset patients

An exploratory analysis was conducted in the very early-onset^31,32^ (VEO; age ≤30 years; *N* = 35) subgroup. Although not statistically significant, these patients showed trends toward more aggressive radiological features, including advanced T stage and mucinous adenocarcinoma (MAC) phenotype (Supplementary Table 9). Notably, within this VEO cohort, our MRI-based risk stratification retained its ability to discriminate recurrence risk (*P* < 0.001), whereas the conventional biomarker, preoperative CEA level, failed to show any prognostic value (Supplementary Fig. 11).

## Discussion

This large, multicenter cohort study demonstrates that EOLARC is defined by a distinct pattern of treatment failure, characterized by a higher risk of local recurrence not fully captured by conventional pathological response assessment. We found that this heightened risk is driven by the disproportionately greater prognostic impact of key pre-treatment MRI features. To translate this insight into clinical practice, we developed the mrTML score, a novel MRI-based tool providing superior risk stratification over existing models. Crucially, our findings challenge the universal role of pCR as the prognostic surrogate, providing an evidence-based framework for risk-adapted therapy in this vulnerable young population.

Our findings lend further weight to the hypothesis that early-onset rectal cancer is not merely an age-shifted version of the disease but represents a biologically distinct entity^10,11^. We found that the distinct biology of EOLARC manifests primarily as a unique pattern of treatment failure, characterized by a higher propensity for local recurrence. This finding is supported by a confluence of evidence throughout our analysis. At baseline, our analysis indicated a trend toward a higher prevalence of MSI in the younger cohort (10.1% vs. 6.2%). Although not statistically significant, likely due to sample size constraints, this observation resonates with emerging literature suggesting a unique molecular landscape inherent to the early-onset phenotype^33^. Furthermore, EOLARC patients presented with a more aggressive radiological profile, including significantly higher rates of features associated with locoregional failure like mrTDs, mrMRF and mrLLN involvement. While these characteristics could be interpreted as signs of more advanced disease, we rigorously balanced baseline tumor burden and clinical staging via PSM to disentangle inherent tumor biology from staging differences^34,35^. The persistence of a higher intrinsic risk of local recurrence in the matched EOLARC cohort implies that this disparity is not driven by anatomical stage alone.

Furthermore, our analysis revealed that this heightened local risk is driven by a disproportionate amplification of the prognostic impact of key MRI risk factors in the younger cohort. The most striking example was mrMRF involvement, which conferred a nearly 2.5-fold greater hazard for disease recurrence in EOLARC patients compared to their late-onset counterparts. While the prognostic significance of MRF involvement is well-established^36,37^, our study is among the first to quantify this dramatic risk amplification in a young population. This age-dependent effect, also observed for mrTDs, provides a powerful explanation for the poorer local control in EOLARC. Crucially, this pattern persisted even within a subgroup restricted to Stage III disease, where high-risk MRI features maintained a stronger association with recurrence in the early-onset group. It suggests that invasive tumors in young patients are biologically more aggressive than morphologically similar tumors in older patients. This concept resonates with emerging evidence on EOLARC’s unique molecular pathways, such as different patterns of chromosomal instability or a higher prevalence of mutations that may potentiate local invasion and resistance to therapy^38,39^.

This inherent biological aggressiveness reveals a critical disconnect between pathological response and clinical outcomes. It is widely accepted that a primary objective of nCRT is to reduce local recurrence, with pCR being its strongest surrogate^28^. However, our study reveals a critical dissociation: despite achieving pCR rates statistically indistinguishable from their late-onset counterparts, patients with EOLARC still experienced a more than twofold higher rate of local failure. Strictly interpreting this disconnect requires a nuanced consideration of pathological limitations. Post-nCRT tumor regression often manifests as tumor fragmentation or isolated tumor cells embedded within extensive fibrosis or mucin pools. Consequently, standard sampling protocols inherently carry a risk of missing these microscopic foci, potentially leading to a false pCR classification. However, our study adhered to rigorous quality control measures, including standardized, extensive sampling protocols and mandatory review by senior gastrointestinal pathologists at tertiary centers. Therefore, technical factors alone cannot fully explain the profound prognostic disparity observed. Instead, the observed recurrences are likely driven by occult minimal residual disease (MRD)^40,41^. It is crucial to recognize that pCR reflects the eradication of the primary tumor but does not necessarily equate to the clearance of systemic micrometastases. High-risk MRI features, particularly mrTDs and mrMRF, are established imaging biomarkers for vascular and lymphatic invasion^36,42^. Their presence implies that micrometastases may have disseminated systemically, persisting below the detection threshold of routine histological examination. This concept aligns with previous studies, such as those by Lord et al. and the OCUM group, which demonstrated that these radiological phenotypes strongly predict distant failure and poor survival independent of standard TNM staging^23,43^. Therefore, for EOLARC, while pCR remains a desirable outcome, its ability to predict long-term freedom from local recurrence appears significantly attenuated. This highlights the urgent need for pre-treatment tools that can identify patients at high intrinsic risk for local failure, independent of their pathological response.

To translate our findings into a clinically applicable tool, we developed the mrTML score. Its construction is methodologically robust, integrating three independent predictors—mrMRF, mrTDs, and mrLLNs—that capture distinct pathways of tumor progression: local infiltration, discontinuous spread, and regional lymphatic dissemination, respectively^22–24^. This multi-faceted approach, combined with the score’s simplicity and high interobserver reliability, ensures its feasibility for widespread clinical implementation. Its clinical value was confirmed by its exceptional prognostic performance, including a high C-index and a steep, dose-response relationship with survival. Furthermore, its ability to predict not just the risk but also the pattern of recurrence—exhibiting over a 44-fold increased risk of local recurrence for the highest-score group—further highlights its potential to guide decisions on local therapy intensification.

The necessity of a tailored model for EOLARC was further highlighted by comparing the mrTML score to a conventional risk model, the mrTEM score, which incorporates mrEMVI instead of mrLLNs^25,27^. In our EOLARC cohort, the mrTML score demonstrated superior prognostic discrimination. We hypothesize that this improved performance is due to our model’s substitution of mrEMVI with mrLLNs. This finding suggests that for younger patients, the regional lymphatic dissemination pathway, directly captured by mrLLNs, may be a more dominant driver of treatment failure than local vascular invasion. This finding not only validates the superiority of the mrTML score but also reinforces our central hypothesis that the drivers of prognosis differ by age, necessitating the development of population-specific tools.

Our pre-treatment risk stratification also provides a new lens through which to interpret pathological response. First, the mrTML score itself predicted therapeutic responsiveness, with low-risk patients demonstrating significantly higher rates of pCR (29.9% vs. 18.9%). This suggests that the high-risk features may be linked to an intrinsic resistance to chemoradiotherapy. Second, and more critically, our analysis demonstrates that pre-treatment MRI risk offers incremental prognostic value beyond pathological response. This was most evident in the pCR subgroup, where high-risk patients still exhibited a greater risk of recurrence. However, we acknowledge that despite the illuminating and clinically relevant of these findings, its associated wide confidence interval requires validation in larger independent cohorts with adequate statistical power. Conversely, in the low-risk group, achieving pCR conferred no significant additional survival advantage. This compelling finding indicates that a favorable pathological response should not be misconstrued as a definitive cure, particularly in patients with high-risk features on baseline imaging.

Building upon these findings, our study provides potential clinical implications for the clinical management of EOLARC, suggesting a risk-adapted therapeutic paradigm guided by the mrTML score. For the substantial proportion of patients identified as low-risk, who demonstrated excellent long-term outcomes irrespective of achieving pCR, our data provide a strong rationale for treatment de-escalation. This supports exploring organ-preservation strategies, such as Watch-and-Wait (W&W) for those who achieve a clinical complete response, or less intensive neoadjuvant regimens in this favorable subgroup, aligning with the principles of ongoing trials like STAR-TREC^44^, with the goal of minimizing long-term treatment-related morbidity. Conversely, for patients stratified into the high-risk group, a multifaceted treatment support approach is warranted. First, in the neoadjuvant setting, our results support the need for prioritizing strategies, such as total neoadjuvant therapy (TNT)^45–47^, to maximize the chances of a favorable pathological response. Second, in the postoperative setting, our stratified analysis revealed that the high-risk subgroup failed to derive a significant survival benefit from adjuvant chemotherapy (HR: 1.92, 95% CI: 0.79–4.67, *P* = 0.14). This observation parallels emerging evidence from clinical trials, which have yet to demonstrate the benefits of adjuvant chemotherapy in rectal cancer^48^. This suggests that high-risk tumors possess intrinsic resistance that cannot be overcome by standard adjuvant regimens. Consequently, escalating standard chemotherapy in this group likely constitutes overtreatment. Instead, intensified follow-up protocols with early salvage interventions may represent a more reasonable and feasible management strategy for these patients^49^. This same principle urges profound caution when contemplating organ-preservation strategies like W&W for any patient initially stratified as high-risk. Since recurrence risk persisted in this subgroup even after achieving pCR, their “complete response” may be less durable than that of their low-risk counterparts. Therefore, if W&W is offered, it should be accompanied by heightened vigilance or intensified surveillance, although we acknowledge this hypothesis requires prospective validation in nonsurgical cohorts. Furthermore, this highlights the potential role of MRD monitoring in the post-operative setting for these high-risk pCR patients to guide decisions on adjuvant therapy or to detect molecular recurrence at the earliest time point.

Our exploratory analysis of the VEO cohort further supports the concept of age-related biological heterogeneity. The observed trend toward more aggressive features in these youngest patients, although not statistically significant due to the small sample size, aligns with reports of distinct molecular drivers in this population, such as a higher frequency of SMAD4 mutations, which are associated with mucinous histology and an aggressive phenotype^50,51^. More importantly, this analysis challenged the utility of conventional biomarkers in the VEO setting. The failure of preoperative CEA to predict outcomes, contrasted with the robust prognostic performance of our MRI-based score, suggests that for this particularly vulnerable group, imaging-based phenotyping is a more reliable prognostic tool than traditional serum markers. While preliminary, these findings highlight the urgent need for dedicated research to establish tailored risk-stratification strategies for the youngest rectal cancer patients.

This study has limitations. First, its retrospective, multicenter design is susceptible to selection bias and treatment heterogeneity, necessitating prospective external validation of the mrTML score. Second, our analysis relied on radiological phenotypes. Given the lack of comprehensive molecular profiling for the entire cohort, the definitive correlations linking aggressive MRI features to underlying molecular drivers require further elucidation through large-scale prospective studies. Finally, the sample size for the exploratory analysis of the VEO subgroup was small. Future studies bridging the gap between imaging, molecular drivers, and pathological verification are a critical next step.

In conclusion, EOLARC is defined by a distinct failure pattern with a higher local recurrence risk, a challenge not adequately captured by pathological response assessment. The MRI-based mrTML score demonstrates potential for pre-treatment risk stratification, creating an essential framework to guide personalized management and improve outcomes for this young patient population.

## Methods

### Study design and participants

We conducted a multicenter, retrospective cohort study following STROBE guidelines. The study was approved by the Institutional Review Board of Guangdong Provincial People’s Hospital (approval number: S2025-175-03) and respective ethics committees of all participating centers, with waivers for informed consent under retrospective study regulations. Clinical trial number: not applicable.

Between September 2009 and December 2020, we enrolled 1289 patients with locally advanced rectal cancer (LARC) from five medical institutions (Supplementary Fig. 1). Patients were stratified into early-onset (EOLARC; <50 years) and late-onset (LOLARC; ≥50 years) cohorts, aligning with age thresholds of major international screening programs^52^. Key inclusion criteria included histologically confirmed rectal adenocarcinoma and defined as LARC on baseline MRI and receipt of standard neoadjuvant chemoradiotherapy followed by total mesorectal excision. Patients managed with a “W&W” strategy or those who received TNT were excluded from this study. Detailed eligibility criteria and participating institutions are provided in Supplementary Note 1.

### Treatment

All LARC patients received standard neoadjuvant chemoradiotherapy consisting of concurrent 5-fluorouracil-based chemotherapy with radiotherapy (50 Gy gross tumor volume/45 Gy clinical target volume delivered over 5 weeks using intensity-modulated techniques), followed by total mesorectal excision 6–8 weeks after completion of radiotherapy. Decisions regarding adjuvant chemotherapy were made by multidisciplinary tumor boards at each institution. Treatment strategies are primarily categorized into three types: oxaliplatin-based combination regimens, irinotecan-based combination regimens, and fluoropyrimidine monotherapy. Complete treatment protocols are detailed in Supplementary Note 2.

### MRI acquisition and interpretation

All patients underwent pretreatment multiparametric rectal MRI across participating centers, including T2-weighted imaging, contrast-enhanced T1-weighted imaging, and diffusion-weighted imaging. Two radiologists (Y.Y. and M.Z., with 5 and 8 years of experience in rectal MRI, respectively) independently assessed MRI biomarkers, including MAC phenotype, mrT stage, mrN stage, mrEMVI, mrTDs, mrMRF, and mrLLNs. Discrepancies were resolved by consensus with a third senior radiologist (W.X., >20 years of MRI experience). Detailed MRI protocols and predefined biomarker assessment criteria are available in the Supplementary Note 3 and Supplementary Table 10.

### Pathological and follow-up assessment

Post-neoadjuvant pathological staging (ypTNM) and TRG were assessed based on the American Joint Committee on Cancer/College of American Pathologists criteria. pCR was stringently defined as the absence of any viable cancer cells in the primary tumor bed and all resected lymph nodes (TRG 0 and ypN0). We also retrieved available molecular data for a subset of 274 patients, as listed in Supplementary Table 1. Patients were followed up every 3–6 months for 5 years, and annually thereafter. The primary endpoint was DFS, defined as the interval from radical resection to first occurrence of locoregional recurrence, distant metastasis, or cancer-related mortality. Secondary endpoints included OS. Detailed endpoint definitions and TRG classifications are available in the Supplementary Note 4.

### Development of the mrTML scoring system

To develop a pre-treatment stratification model tailored for EOLARC patients, we conducted prognostic factor analysis within this specific cohort. Candidate prognostic variables were initially screened using univariable Cox regression analysis. Variables with *P* < 0.05 were subsequently entered into a multivariable Cox regression model to identify independent predictors. This identified mrMRF involvement, presence of mrTDs, and mrLLNs metastasis as the most powerful independent predictors of DFS (Supplementary Table 3). To create a clinically practical tool, we developed the mrTML score by integrating these three prognostic biomarkers. Based on the principle of parsimony and similar weighting in previous MRI-based models^25,27^, each predictor was treated as a binary variable and assigned a value of 1 if present and 0 if absent. The final mrTML score was calculated as the unweighted sum of these points, yielding a total score ranging from 0 to 3. Interobserver reliability was assessed using Cohen’s kappa coefficient among radiologists with varying experience levels.

### Statistical analysis

Quantitative data were presented as median with interquartile range and compared using Student’s *t* test or Mann–Whitney U-test based on normality testing. Categorical variables were analyzed via chi-square test or Fisher’s exact test. To mitigate confounding bias, PSM was implemented using one-to-one nearest-neighbor matching with caliper adjustment, based on gender, preoperative CEA level, cTNM stage, tumor location, mucinous adenocarcinoma, and MRI parameters (mrT stage, mrN stage, mrEMVI, mrTDs, mrMRF, and mrLLNs). Balance was assessed using SMD. Survival outcomes were analyzed using Cox regression models, with univariate screening (*P* < 0.05) followed by multivariable analysis for independent predictors in EOLARC patients. Kaplan–Meier curves with log-rank tests visualized survival differences. Model performance was evaluated using Harrell’s concordance index (C-index) from 1000 bootstrap resamples. All analyses were conducted using R software version 4.3.3.

## Supplementary information

Supplementary Information

## Data Availability

The data analyzed during the current study are available from the corresponding author on reasonable request.
