# Supplementary material for: Higher local recurrence as the distinct failure pattern in early-onset rectal cancer: a tailored MRI score to guide therapy
Source: NPJ Precis Oncol. 2025 Dec 24;10:44. doi: 10.1038/s41698-025-01244-6 (PMC12847736; doi:10.1038/s41698-025-01244-6)
Supplement: Supplementary file 1 — Supplementary Information [file 41698_2025_1244_MOESM1_ESM.pdf]

Higher Local Recurrence as the Distinct Failure Pattern in Early-Onset Rectal Cancer: A Tailored MRI Score to Guide Therapy

Supplementary materials

Table of contents

Supplementary Note.

Supplementary Table 1. Molecular Characteristics in Patients with Early-onset and Late-onset Locally Advanced Rectal Cancer.

Supplementary Table 2. Epidemiologic and Tumor Characteristics Before and After Propensity Score Matching in Early- and Late-Onset Rectal Cancer.

Supplementary Table 3. Univariable and Multivariable Cox Regression Analyses for DFS and OS in EOLARC.

Supplementary Table 4. Demographic and Tumor Characteristics by MRI Markers in EOLARC.

Supplementary Table 5. Inter-reader Agreement for MRI Marker Status and mrTML Score in 100 Patients.

Supplementary Table 6. Tumor Characteristics by mrTML Score in EOLARC.

Supplementary Table 7. Univariable and Multivariable Cox Regression Analyses for LR and DM in EOLARC.

Supplementary Table 8. Concordance Index Values of Clinical and Imaging Factors for Predicting DFS and OS.

Supplementary Table 9. Epidemiologic and Tumor Characteristics in Very Early-Onset vs Early-Onset Rectal Cancer.

Supplementary Table 10. MRI Acquisition Parameters Across Participating Centers.

Supplementary Figure 1. Flowchart of the Patient Recruitment Process.

Supplementary Figure 2. Kaplan-Meier Survival Analysis in the Propensity Score-Matched Cohort.

Supplementary Figure 3. Comparison of Prognostic Factors Between Early-Onset and Late-Onset Locally Advanced Rectal Cancer in Stage III After Propensity Score Matching.

Supplementary Figure 4. Prognostic Impact of MRI Biomarkers in EOLARC.

Supplementary Figure 5. Prognostic Impact of mrTML Score in EOLARC.

Supplementary Figure 6. Prognostic Performance for Predicting Overall Survival (OS) in EOLARC.

Supplementary Figure 7. Kaplan-Meier Curves of Overall Survival (OS) Stratified by MRI-Based Risk Stratification in Early-Onset Locally Advanced Rectal Cancer.

Supplementary Figure 8. Association Between MRI-Based Risk Stratification and Treatment Response in EOLARC.

Supplementary Figure 9. Prognostic Stratification by Pathological Response Status in MRI-Based Risk Subgroups.

Supplementary Figure 10. Impact of MRI Risk Stratification and Adjuvant Chemotherapy on Disease-Free Survival (DFS) in Early-Onset Locally Advanced Rectal Cancer.

Supplementary Figure 11. Prognostic Impact of CEA Level and MRI-Based Risk Stratification in Very Early-Onset (Age ≤30 Years) Locally Advanced Rectal Cancer.

Supplementary Note 1. Patient cohort and eligibility criteria

Patients diagnosed with locally advanced rectal cancer (LARC) between September 2009 and December 2020 were retrospectively collected from five medical institutions, including Guangdong Provincial People's Hospital (GDPH; Guangzhou, China), Sun Yat-sen University Cancer Center (SYSUCC; Guangzhou, China), the Sixth Affiliated Hospital of Sun Yat-sen University (SYS6; Guangzhou, China), Shanxi Provincial Cancer Hospital (SXCH; Taiyuan, China), and Yunnan Provincial Cancer Hospital (YNCH; Kunming, China). Patient enrollment at each center was determined by the total eligible population during the specified timeframe.

Inclusion criteria were as follows: (a) Patients aged ≥18 years with histologically confirmed rectal adenocarcinoma via electronic colonoscopy with biopsy and defined as LARC (cT3-4/N0-2, EMVI+, or any T/N1-2, and M0) based on pelvic magnetic resonance imaging (MRI) findings;[1] (b) All patients underwent standard total mesorectal excision (TME) surgery after receiving standard neoadjuvant chemoradiotherapy (nCRT) regimen; (c) Tumor regression grade (TRG) and pathological response (pathological complete response [pCR] vs. non-pCR) assessed by experienced pathologists postoperatively; (d) Availability of high-quality, pre-treatment MRI scans, including T2-weighted imaging (T2WI), contrast-enhanced T1-weighted imaging (CE-T1WI), and diffusion-weighted imaging (DWI), all acquired within 2 weeks prior to nCRT initiation; (e) No metastasis or other tumor events occurred during nCRT and operation.

Exclusion criteria were as follows: (a) History of other malignancies, prior chemotherapy, radiotherapy, or surgical interventions; (b) Receipt of total neoadjuvant therapy, incomplete or nonstandard nCRT, or receipt of chemotherapy or radiotherapy alone; (c) Management with a 'Watch-and-Wait' strategy (i.e., absence of radical resection) or unavailable pathological assessment of resected specimens; (d) Inaccessible MRI data or insufficient quality of MRI images for assessment (i.e., motion artifacts).

Clinicopathological variables including age at diagnosis, sex, pretreatment carcinoembryonic antigen (CEA), and clinical TNM stage were systematically extracted from institutional electronic health record systems. Pretreatment CEA measurements followed standardized protocols (blood collection <14 days before treatment initiation), with levels <5 ng/mL classified as negative and ≥5 ng/mL as positive.

Supplementary Note 2. Treatment protocols

Neoadjuvant chemoradiotherapy treatment for LARC patients

All LARC patients included in this study underwent a standardized neoadjuvant chemoradiotherapy (nCRT) protocol. The protocol consisted of radiotherapy administered concurrently with a 5-fluorouracil-based chemotherapy regimen (delivered orally or intravenously). The neoadjuvant radiotherapy was delivered at 25 fractions of 2 Gy (gross tumor volume, GTV) and 1.8 Gy (clinical target volume, CTV) radiation, using Intensity-Modulated Radiation Therapy (IMRT) or Volumetric Modulated Arc Therapy (VMAT) technique. A total dose of 50 Gy (GTV)/45 Gy (CTV) radiation was administered at 2.0 Gy/1.8 Gy per fraction, over a period (weekdays, day 1 to day 5) of 5 weeks. The TME surgery was performed

within 6-8 weeks after the completion of nCRT, based on standard TME operation protocol.

Postoperative chemoradiotherapy treatment for LARC patients

Decisions regarding adjuvant chemotherapy were made by multidisciplinary tumor boards at each institution based on a comprehensive assessment of pathological staging (ypTNM), tumor regression grade, patient performance status, and individual preferences. Treatment duration typically ranged from 3 to 6 months according to institutional protocols and patient tolerance. Specifically, Treatment strategies were stratified into oxaliplatin-based combination therapies, irinotecan-based combinations, and fluoropyrimidine monotherapy. The most common regimens were FOLFOX and CAPOX (XELOX), which represented the standard of care for most eligible patients. FOLFIRI served as a primary alternative for patients with contraindications to oxaliplatin. Fluoropyrimidine monotherapy (typically with capecitabine) was reserved for patients unable to tolerate combination chemotherapy or for select cases of lower-risk disease.

Supplementary Note 3. Evaluation criteria and process for MRI biomarkers

(a) mrT stage

MRI-identified T and N stage were evaluated according to the eighth edition of the American Joint Committee on Cancer (AJCC) TNM staging system. Stage T1/T2 shows intact hypointense line surrounding the rectum on MRI. T3 stage shows the interruption of the hypointense muscularis propria with specular or nodular extension of tumor signal beyond the rectal wall into the mesorectal fat. T3 substage was determined as: T3a (extramural depth of tumor invasion <1 mm), T3b (extramural depth, 1-5 mm), T3c (extramural depth, 5-15 mm) and T3d (extramural depth >15 mm). T4a stage tumors invade the peritoneum or peritoneal reflection, while T4b invades organs or structures outside the rectal mesorectum.[2]

(b) mrN stage

N0, no suspicious lymph nodes; N1, 1-3 suspicious lymph nodes/no suspicious lymph nodes but tumor deposits; N2, ≥ 4 suspicious lymph nodes.[3]

The criteria for suspicion were as follows: 1) Short axis diameter ≥ 9 mm; 2) Short axis diameter 5-8 mm and ≥ 2 morphologically suspicious characteristics; 3) Short axis diameter <5 mm and 3 morphologically suspicious characteristics; 4) Mucinous lymph nodes (any size). Malignant characteristics including indistinct border, heterogeneous signal, and round shape.

(c) MRI-identified EMVI

Vessels adjacent to the tumor exhibit intermediate signal intensity on MRI, accompanied by either mild expansion of vessel contour and caliber, or obvious abnormal vessel outlines and/or nodular dilatation, indicating tumor invasion.[4]

(d) MRI-identified TDs

Identified as irregular nodules within the mesorectum that directly interrupt the course of veins but are discontinuous from the primary tumor. mrTDs can be distinguished from lymph node metastases as they cannot be separated from the vein when assessed on two orthogonal views and tend to taper into the vein rather than being alongside the vein and forming an acute angle.[5]

(e) MRI-identified MRF involvement

The shortest distance from the outermost edge of the major tumor extension, EMVI, tumor deposits,

or suspicious lymph node to the mesenteric fascia. >1 mm is considered negative and ≤1 mm is considered positive.[6]

(f) MRI-identified LLNs

Assessment of LLNs status was based on the largest LLNs in external iliac region, obturator region, and internal iliac region identified on pretreatment MRI. Suspicious LLNs were defined as those with a short axis ≥7 mm, according to recently suggested criteria.[7]

(g) MAC phenotype

Identified as higher T2 signal intensity than that of the surrounding fat occupying more than half of the tumor volume.[8]

Two radiologists (Y.Y. and M.Z., with 5 and 8 years of experience in rectal MRI, respectively) underwent standardized training under the guidance of senior radiologist prior to image evaluation to harmonize interpretation criteria. They independently assessed the presence of key MRI biomarkers on de-identified baseline MRI scans, including MAC phenotype, mrT stage, mrN stage, mrEMVI, mrTDs, mrMRF, and mrLLNs, based on predefined radiologic criteria. Any discrepancies between the two readers were resolved by consensus in consultation with a senior radiologist (W.X., >20 years of MRI experience). All readers were blinded to clinical, pathological, and outcome data throughout the evaluation process to minimize interpretation bias. Image analysis was conducted using RadiAnt DICOM Viewer (version 5.0.1).

To assess the reproducibility of the critical MRI biomarkers, a set of 100 consecutive cases was independently reviewed for mrTDs, mrMRF, and mrLLNs status by three radiologists representing different experience levels: junior (<5 years), intermediate (5-10 years), and senior (>15 years).

Supplementary Note 4. Endpoint definitions, TRG classifications and detailed statistical methods

Patient follow-up

The primary endpoint was DFS, defined as the interval from radical resection to the first occurrence of locoregional recurrence, distant metastasis, or cancer-related mortality. Locoregional recurrence (LR) was strictly defined as tumor reappearance at the primary site or within the pelvic anatomical compartment, while distant metastasis (DM) was defined as tumor spread to distant organs/tissues outside the pelvis. Definitive diagnosis of recurrence or metastasis was based on radiological evidence and/or histopathological confirmation. Secondary endpoints included overall survival (OS), calculated from surgery to cancer-related death. Patients alive without event occurrence were censored at the last validated follow-up contact.

Pathological treatment response evaluation

The TME surgery was performed 6 to 8 weeks following the completion of radiotherapy. Residual tumor evaluation on hematoxylin and eosin (H&E)-stained surgical specimens was conducted according to a standardized reporting protocol at each participating institution. Treatment response was stratified using the four-tier tumor regression grading (TRG) system proposed by the American Joint Committee on Cancer/College of American Pathologists (AJCC/CAP), based on the extent of residual tumor cells: TRG

0 (complete response), indicating no viable cancer cells; TRG 1 (moderate response), with only small clusters or single cancer cells remaining; TRG 2 (minimal response), showing residual tumor amid predominant fibrosis; and TRG 3 (poor response), characterized by extensive residual tumor with minimal or no evidence of tumor cell death [1]. TRG data were retrospectively collected from five hospitals. Patients classified as TRG 0 with negative lymph node metastasis were designated as pathological complete response (pCR) cases, whereas those with TRG 1-3 or positive lymph node metastasis were categorized as non-pCR cases.

Detailed Statistical Methods

All statistical analyses were performed using R Studio software version 4.3.3 (https://www.rstudio.com/). Quantitative variables were presented as median with interquartile range (IQR) and assessed for normality using the Shapiro-Wilk test ($P>0.05$ threshold), with subsequent comparisons performed using Student's t-test for normally distributed data or Mann-Whitney U-test for non-parametric data. Categorical variables were described as frequencies and percentages, with between-group comparisons conducted using chi-square tests or Fisher's exact tests when expected cell counts were less than 5. Propensity score matching was implemented using a nearest-neighbor matching algorithm with caliper adjustment to create balanced EOLARC and LOLARC cohorts, matching on covariates including gender, preoperative CEA level, cTNM stage, tumor location, mucinous adenocarcinoma status, mrT stage, mrN stage, mrEMVI, mrTDs, mrMRF, and mrLLNs, with balance assessed using standardized mean differences (SMD). Univariate Cox regression analysis was performed to identify candidate predictors of overall survival (OS) and disease-free survival (DFS), with variables showing $P<0.05$ entered into multivariable Cox models to identify independent prognostic factors specifically within the EOLARC cohort. Treatment response rates between groups were compared using chi-square tests, and survival disparities were visualized using Kaplan-Meier curves with log-rank tests, with hazard ratios (HRs) and 95% confidence intervals (CIs) calculated to quantify prognostic discrimination. Model performance was quantified using Harrell's concordance index (C-index) derived from 1000 bootstrap resamples. Interobserver agreement for MRI assessments was evaluated using Cohen's kappa coefficient with the following interpretation: 0-0.20 = slight, 0.21-0.40 = fair, 0.41-0.60 = moderate, 0.61-0.80 = substantial, and 0.81-1.00 = excellent agreement, with linear weighted kappa tests applied for mrTDs, mrMRF, and mrLLNs variables, and squared weighted kappa tests used for the mrTML score assessment.

Supplementary References

1. Glynne-Jones R, Wyrwicz L, Tiret E, et al. Rectal cancer: ESMO Clinical Practice Guidelines for diagnosis, treatment and follow-up. Ann Oncol Off J Eur Soc Med Oncol 2017;28:iv22-iv40.

2. Cho SH, Kim SH, Bae JH, et al. Prognostic stratification by extramural depth of tumor invasion of primary rectal cancer based on the Radiological Society of North America proposal. AJR Am J Roentgenol 2014;202:1238-1244.

3. Beets-Tan RGH, Lambregts DMJ, Maas M, et al. Magnetic resonance imaging for clinical management of rectal cancer: Updated recommendations from the 2016 European Society of Gastrointestinal and Abdominal Radiology (ESGAR) consensus meeting. Eur Radiol 2018;28:1465-

1475.

4. Smith NJ, Barbachano Y, Norman AR, et al. Prognostic significance of magnetic resonance imaging-detected extramural vascular invasion in rectal cancer. Br J Surg 2008;95:229-236.

5. Lord AC, D'Souza N, Shaw A, et al. MRI-Diagnosed Tumor Deposits and EMVI Status Have Superior Prognostic Accuracy to Current Clinical TNM Staging in Rectal Cancer. Ann Surg 2022;276:334-344.

6. Taylor FGM, Quirke P, Heald RJ, et al. Preoperative Magnetic Resonance Imaging Assessment of Circumferential Resection Margin Predicts Disease-Free Survival and Local Recurrence: 5-Year Follow-Up Results of the MERCURY Study. J Clin Oncol 2014;32:34-43.

7. Ogura A, Konishi T, Cunningham C, et al. Neoadjuvant (Chemo)radiotherapy With Total Mesorectal Excision Only Is Not Sufficient to Prevent Lateral Local Recurrence in Enlarged Nodes: Results of the Multicenter Lateral Node Study of Patients With Low cT3/4 Rectal Cancer. J Clin Oncol Off J Am Soc Clin Oncol 2019;37:33-43.

8. Park SH, Lim JS, Lee J, et al. Rectal Mucinous Adenocarcinoma: MR Imaging Assessment of Response to Concurrent Chemotherapy and Radiation Therapy-A Hypothesis-generating Study. Radiology 2017;285:124-133.

Supplementary Table 1. Molecular Characteristics in Patients with Early-onset and Late-onset Locally Advanced Rectal Cancer.

Characteristics	All	EOLARC	LOLARC	P value
**MMR**				0.37
MSS	254 (92.7)	71 (89.9)	183 (93.8)	
MSI	20 (7.3)	8 (10.1)	12 (6.2)	
**NRAS**				1.00
Wild-type	144 (52.6)	38 (48.1)	106 (54.4)	
Mutant	3 (1.1)	1 (1.3)	2 (1.0)	
NA	127 (46.4)	40 (50.6)	87 (44.6)	
**HER2**				0.16
Negative (0/1+)	171 (62.4)	55 (69.6)	116 (59.5)	
Equivocal (2+)	44 (16.1)	8 (10.1)	36 (18.5)	
Positive (3+)	6 (2.2)	1 (1.3)	5 (2.6)	
NA	53 (19.3)	15 (19.0)	38 (19.5)	
**KRAS**				0.47
Wild-type	119 (43.4)	29 (36.7)	90 (46.2)	
Mutant	27 (9.9)	9 (11.4)	18 (9.2)	
NA	128 (46.7)	41 (51.9)	87 (44.6)	
**P53**				0.30
Wild-type	42 (15.3)	6 (7.6)	36 (18.5)	
Mutant	65 (23.7)	16 (20.3)	49 (25.1)	
NA	167 (60.9)	57 (72.2)	110 (56.4)	
**Ki67**				0.51
≤40	145 (52.9)	45 (57.0)	100 (51.3)	
>40	81 (29.6)	21 (26.6)	60 (30.8)	
NA	48 (17.5)	13 (16.5)	35 (17.9)	
**CDX2**				1.00
Negative	118 (43.1)	42 (53.2)	76 (39.0)	
Positive	4 (1.5)	1 (1.3)	3 (1.5)	
NA	152 (55.5)	36 (45.6)	116 (59.5)	

Unless otherwise indicated, data are No. (%).
NA, not available. P-values were calculated excluding NA categories.

Supplementary Table 2. Epidemiologic and Tumor Characteristics Before and After Propensity Score Matching in Early- and Late-Onset Rectal Cancer.

Characteristics	All	After PSM matching[a]		*P*	SMD
		EOLARC	LOLARC		
	N = 696	N = 348	N = 348		
Age, years[b]	49.5 [43-61]	43 [37-46]	61 [55-65]	<0.001	NA
Gender					
Male	454 (65.2)	228 (65.5)	226 (64.9)	0.94	0.01
Female	242 (34.8)	120 (34.5)	122 (35.1)		
CEA level[c]					
Normal	451 (64.8)	226 (64.9)	225 (64.7)	1.00	0.01
Abnormal	245 (35.2)	122 (35.1)	123 (35.3)		
Clinical stage					
II	97 (13.9)	50 (14.4)	47 (13.5)	0.83	0.03
III	599 (86.1)	298 (85.6)	301 (86.5)		
Location					
Low	346 (49.7)	174 (50.0)	172 (49.4)	0.94	0.01
Middle-High	350 (50.3)	174 (50.0)	176 (50.6)		
MAC					
Negative	663 (95.3)	331 (95.1)	332 (95.4)	1.00	0.01
Positive	33 (4.7)	17 (4.9)	16 (4.6)		
mrT stage					
T2-T3a/b	399 (57.3)	197 (56.6)	202 (58.1)	0.76	0.03
T3c/d-T4	297 (42.7)	151 (43.4)	146 (41.9)		
mrN stage					
N0	259 (37.2)	128 (36.8)	131 (37.6)	0.88	0.02
N1-N2	437 (62.8)	220 (63.2)	217 (62.4)		
mrTDs status					
Negative	550 (79.0)	271 (77.9)	279 (80.2)	0.53	0.06
Positive	146 (21.0)	77 (22.1)	69 (19.8)		
mrEMVI status					
Negative	501 (72.0)	243 (69.8)	258 (74.1)	0.24	0.10
Positive	195 (28.0)	105 (30.2)	90 (25.9)		
mrMRF involvement					
Negative	485 (69.7)	237 (68.1)	248 (71.3)	0.41	0.07
Positive	211 (30.3)	111 (31.9)	100 (28.7)		
mrLLNs status					
Negative	611 (87.8)	304 (87.4)	307 (88.2)	0.82	0.03
Positive	85 (12.2)	44 (12.6)	41 (11.8)		
R0 resection status					
R0 (negative margin)	576 (82.8)	291 (83.6)	289 (83.1)	0.62	NA
R1 (positive margin)	3 (0.4)	1 (0.3)	2 (0.6)		

NA	117 (16.8)	56 (16.1)	57 (16.4)		
Surgical procedure type[d]					
Sphincter-Preserving Surgery	407 (58.5)	203 (58.3)	204 (58.6)	0.95	NA
Abdominoperineal Resection	176 (25.3)	89 (25.6)	87 (25.0)		
NA	113 (16.2)	56 (16.1)	57 (16.4)		
Adjuvant chemotherapy					
Yes	214 (30.7)	113 (32.5)	101 (29.0)	0.03	NA
No	50 (7.2)	17 (4.9)	33 (9.5)		
NA	432 (62.1)	218 (62.6)	214 (61.5)		
Local recurrence					
No	560 (80.5)	275 (79.0)	285 (81.9)	0.03	NA
Yes	23 (3.3)	17 (4.9)	6 (1.7)		
NA	113 (16.2)	56 (16.1)	57 (16.4)		
Distant metastasis					
No	496 (71.3)	251 (72.1)	245 (70.4)	0.63	NA
Yes	87 (12.5)	41 (11.8)	46 (13.2)		
NA	113 (16.2)	56 (16.1)	57 (16.4)		
Total recurrence					
No	563 (80.9)	286 (82.2)	277 (79.6)	0.44	NA
Yes	133 (19.1)	62 (17.8)	71 (20.4)		

Unless otherwise indicated, data are No. (%).

[a]Propensity score matching covariates included gender, preoperative CEA level, cTNM stage, tumor location, MAC, mrT stage, mrN stage, mrEMVI, mrTDs, mrMRF, and mrLLNs; these were used for 1:1 nearest-neighbor matching with caliper adjustment.

[b]Data are medians, with IQRs in parentheses.

[c]Normal values for CEA level range from 0 to 5 ng/mL.

[d]Sphincter-preserving surgery includes Dixon, Parks, Intersphincteric Resection, Bacon, and TaTME.

Abbreviations: EOLARC, early-onset locally advanced rectal cancer; LOLARC, late-onset locally advanced rectal cancer; PSM, propensity score matching; SMD, standardized mean difference; CEA, carcinoembryonic antigen; cTNM, clinical tumor-node-metastasis stage; MAC, mucinous adenocarcinoma; mr, magnetic resonance; TDs, tumor deposits; EMVI, extramural vascular invasion; MRF, mesorectal fascia; LLNs, lateral lymph nodes.

Supplementary Table 3. Univariable and Multivariable Cox Regression Analyses for DFS and OS in EOLARC.

	Disease-free survival				Overall survival			
	Univariate analysis		Multivariate analysis		Univariate analysis		Multivariate analysis	
	HR (95% CI)	*P*	AHR (95% CI)	*P*	HR (95% CI)	*P*	AHR (95% CI)	*P*
Age	1.00 (0.97-1.04)	0.95			0.95 (0.91-0.99)	0.02		
Gender								
Male	Reference				Reference			
Female	0.96 (0.59-1.54)	0.85			1.31 (0.67-2.54)	0.43		
CEA level[a]								
Normal	Reference				Reference		Reference	
Abnormal	1.72 (1.09-2.71)	0.02			2.72 (1.41-5.26)	<0.01	1.92 (0.98-3.75)	0.06
cTNM stage								
II	Reference				Reference			
III	2.30 (0.93-5.70)	0.07			0.98 (0.38-2.53)	0.97		
Location								
Low	Reference				Reference			
Middle & High	0.85 (0.54-1.33)	0.48			0.97 (0.50-1.86)	0.92		
MAC								
Negative	Reference				Reference		Reference	
Positive	2.37 (1.25-4.50)	0.01			3.45 (1.51-7.88)	<0.01	2.18 (0.93-5.09)	0.07
mrT stage								
T2-T3a/b	Reference		Reference		Reference		Reference	
T3c/d-T4	4.60 (2.71-7.83)	<0.001	1.91 (1.00-3.63)	0.05	5.99 (2.62-13.68)	<0.001	2.36 (0.88-6.28)	0.09
mrN stage								
N0	Reference				Reference			
N1-2	3.20 (1.78-5.73)	<0.001			3.10 (1.35-7.10)	0.01		
mrEMVI status								
Negative	Reference				Reference			
Positive	3.39 (2.15-5.34)	<0.001			2.85 (1.48-5.49)	<0.01		
mrTDs status								
Negative	Reference		Reference		Reference		Reference	
Positive	5.97 (3.77-9.45)	<0.001	3.43 (2.11-5.58)	<0.001	5.57 (2.88-10.78)	<0.001	3.02 (1.49-6.13)	<0.01
mrMRF status								
Negative	Reference		Reference		Reference		Reference	
Positive	5.29 (3.27-8.56)	<0.001	2.12 (1.17-3.84)	0.01	6.44 (3.11-13.37)	<0.001	2.23 (0.91-5.47)	0.08
mrLLNs status								
Negative	Reference		Reference		Reference			
Positive	4.84 (3.04-7.72)	<0.001	3.16 (1.96-5.09)	<0.001	2.47 (1.19-5.12)	0.02		

[a]The normal values for CEA level range from 0 to 5 ng/ml.

Abbreviation: EOLARC, early-onset locally advanced rectal cancer; HR, hazard ratio; AHR, adjusted hazard ratio; CI, confidence interval; CEA, carcinoembryonic antigen; cTNM stage, clinical tumor-node-metastasis stage; MAC, mucinous adenocarcinoma; mr, magnetic resonance; EMVI, extramural vascular invasion; TDs, tumor deposits; MRF, mesorectal fascia; LLNs, lateral lymph nodes.

Supplementary Table 4. Demographic and Tumor Characteristics by MRI Markers in EOLARC.

	mrTDs		*P*	mrMRF		*P*	mrLLNs		*P*
**Features**	**Negative**	**Positive**		**Negative**	**Positive**		**Negative**	**Positive**	
Age[a], years	43 [38-47]	41 [35-46]	0.11	43 [38-46]	42 [36-46]	0.21	43 [37-46]	42 [38-47]	0.72
Gender									
Male	193 (65.0)	56 (62.2)	0.72	175 (66.3)	74 (60.2)	0.29	217 (65.8)	32 (56.1)	0.21
Female	104 (35.0)	34 (37.8)		89 (33.7)	49 (39.8)		113 (34.2)	25 (43.9)	
CEA level[b]									
Normal	205 (69.0)	50 (55.5)	0.03	185 (70.1)	70 (56.9)	0.02	222 (67.3)	33 (57.9)	0.22
Abnormal	92 (31.0)	40 (44.5)		79 (29.9)	53 (43.1)		108 (32.7)	24 (42.1)	
cTNM stage									
II	49 (16.5)	4 (4.5)	0.01	42 (15.9)	11 (8.9)	0.09	53 (16.1)	0 (0.0)	<0.01
III	248 (83.5)	86 (95.5)		222 (84.1)	112 (91.1)		277 (83.9)	57 (100.0)	
Location									
Low	161 (54.2)	32 (35.5)	<0.01	131 (49.6)	62 (50.4)	0.97	158 (47.9)	35 (61.4)	0.08
Middle & High	136 (45.8)	58 (64.5)		133 (50.4)	61 (49.6)		172 (52.1)	22 (38.6)	
MAC									
Negative	276 (92.9)	82 (91.1)	0.73	251 (95.1)	107 (87.0)	0.01	310 (93.9)	48 (84.2)	0.02
Positive	21 (7.1)	8 (8.9)		13 (4.9)	16 (13.0)		20 (6.1)	9 (15.8)	
mrT stage									
T2–T3a/b	186 (62.6)	20 (22.2)	<0.001	189 (71.6)	17 (13.8)	<0.001	188 (57.0)	18 (31.6)	<0.001
T3c/d-T4	111 (37.4)	70 (77.8)		75 (28.4)	106 (86.2)		142 (43.0)	39 (68.4)	
mrN stage									
N0	135 (45.5)	8 (8.9)	<0.001	123 (46.6)	20 (16.3)	<0.001	139 (42.1)	4 (7.0)	<0.001
N1-N2	162 (54.5)	82 (91.1)		141 (53.4)	103 (83.7)		191 (57.9)	53 (93.0)	
mrEMVI status									
Negative	247 (83.2)	22 (24.4)	<0.001	214 (81.1)	55 (44.7)	<0.001	237 (71.8)	32 (56.1)	0.03
Positive	50 (16.8)	68 (75.6)		50 (18.9)	68 (55.3)		93 (28.2)	25 (43.9)	
TRG[c]									
0	82 (27.9)	18 (20.0)		79 (30.3)	21 (17.0)		85 (25.9)	15 (26.8)	
1	74 (25.2)	29 (32.2)	0.07	68 (26.1)	35 (28.5)	0.01	88 (26.8)	15 (26.8)	0.62
2	118 (40.1)	31 (34.5)		98 (37.5)	51 (41.5)		130 (39.7)	19 (33.9)	
3	20 (6.8)	12 (13.3)		16 (6.1)	16 (13.0)		25 (7.6)	7 (12.5)	

Unless otherwise indicated, data are No. (%). *P* values indicate comparisons between the EOLARC and LOLARC groups; *P*<.05 was considered statistically significant.

[a]Data are medians, with IQRs in parentheses.

[b]Normal values for CEA level range from 0 to 5 ng/mL.

[c]Three cases could not be assessed for TRG due to insufficient archival material.

Abbreviations: EOLARC, early-onset locally advanced rectal cancer; LOLARC, late-onset locally advanced rectal cancer; CEA, carcinoembryonic antigen; cTNM, clinical tumor-node-metastasis stage; MAC, mucinous adenocarcinoma; mr, magnetic resonance;

TDs, tumor deposits; EMVI, extramural vascular invasion; MRF, mesorectal fascia; LLNs, lateral lymph nodes; TRG, tumor regression grade

Supplementary Table 5. Interreader Agreement for MRI Marker Status and mrTML Score in 100 Patients.

	Cohen's kappa[a]			
	**mrTDs[b]**	**mrMRF**	**mrLLNs**	**mrTML**
R1/R2[c]	0.63	0.69	0.76	0.84
R1/R3	0.71	0.69	0.89	0.82
R2/R3	0.77	0.63	0.80	0.76

[a]Linear-weighted kappa was used for all measures except mrTML, for which squared-weighted kappa was used.

[b]All MRI markers (mrTDs, mrMRF, mrLLNs) were assessed for status only (present vs absent), not for stage.

[c]R1 indicates junior radiologists; R2, intermediate radiologists; R3, senior radiologists. Interpretation of kappa: 0.81-1.00, excellent agreement; 0.61-0.80, substantial; 0.41-0.60, moderate; 0.21-0.40, fair; 0-.20, poor or no agreement.

Abbreviations: MRI, magnetic resonance imaging; mr, magnetic resonance (MRI-defined); TDs, tumor deposits; MRF, mesorectal fascia; LLNs, lateral lymph nodes; mrTML, composite score integrating mrTDs, mrMRF, and mrLLNs status.

Supplementary Table 6. Tumor Characteristics by mrTML Score in EOLARC.

**Features**	**mrTML score =0**	**mrTML score =1**	**mrTML score =2**	**mrTML score =3**	***P***
Age[a], years	43 [38-46]	43 [36-47]	41 [34.5-46]	41 [36-45.5]	0.21
Gender					
Male	142 (67.3)	61 (60.4)	37 (66.1)	9 (47.4)	0.27
Female	69 (32.7)	40 (39.6)	19 (33.9)	10 (52.6)	
CEA level[b]					
Normal	151 (71.6)	64 (63.4)	31 (55.4)	9 (47.4)	0.03
Abnormal	60 (28.4)	37 (36.6)	25 (44.6)	10 (52.6)	
cTNM stage					
II	40 (19.0)	11 (10.9)	2 (3.6)	0 (0.0)	<0.01
III	171 (81.0)	90 (89.1)	54 (96.4)	19 (100.0)	
Location					
Low	105 (49.8)	57 (56.4)	21 (37.5)	10 (52.6)	0.16
Middle & High	106 (50.2)	44 (43.6)	35 (62.5)	9 (47.4)	
MAC					
Negative	202 (95.7)	91 (90.1)	49 (87.5)	16 (84.2)	0.05
Positive	9 (4.3)	10 (9.9)	7 (12.5)	3 (15.8)	
mrT stage					
T2–T3a/b	162 (76.8)	34 (33.7)	9 (16.1)	1 (5.3)	<0.001
T3c/d-T4	49 (23.2)	67 (66.3)	47 (83.9)	18 (94.7)	
mrN stage					
N0	116 (55.0)	22 (21.8)	5 (8.9)	0 (0.0)	<0.001
N1-N2	95 (45.0)	79 (78.2)	51 (91.1)	19 (100.0)	
mrEMVI status					
Negative	183 (86.7)	68 (67.3)	13 (23.2)	5 (26.3)	<0.001
Positive	28 (13.3)	33 (32.7)	43 (76.8)	14 (73.7)	
TRG[c]					
0	64 (30.6)	21 (21.0)	12 (21.4)	3 (15.8)	
1	51 (24.4)	30 (30.0)	17 (30.4)	5 (26.3)	0.23
2	82 (39.2)	40 (40.0)	20 (35.7)	7 (36.8)	
3	12 (5.8)	9 (9.0)	7 (12.5)	4 (21.1)	

Unless otherwise indicated, data are No. (%).
[a]Data are medians, with IQRs in parentheses.
[b]The normal values for CEA level range from 0 to 5 ng/ml.
[c]Three cases could not be assessed for TRG due to lack of archival material.
Abbreviation: EOLARC, early-onset locally advanced rectal cancer; mrTML score, the composite score integrating mrTDs, mrMRF, and mrLLNs status; CEA, carcinoembryonic antigen; cTNM stage, clinical tumor-node-metastasis stage; MAC, mucinous adenocarcinoma; mr, magnetic resonance; EMVI, extramural vascular invasion; TRG, tumor regression grade.

Supplementary Table 7. Univariable and Multivariable Cox Regression Analyses for LR and DM in EOLARC.

	Local recurrence				Distant metastasis			
	Univariate analysis		Multivariate analysis		Univariate analysis		Multivariate analysis	
	HR (95% CI)	*P*	AHR (95% CI)	*P*	HR (95% CI)	*P*	AHR (95% CI)	*P*
Age	0.98 (0.92-1.04)	0.57			1.02 (0.98-1.07)	0.32		
Gender								
Male	Reference				Reference			
Female	1.04 (0.42-2.61)	0.93			0.77 (0.42-1.43)	0.41		
CEA level[a]								
Normal	Reference				Reference			
Abnormal	2.20 (0.92-5.31)	0.08			1.96 (1.12-3.43)	0.02		
cTNM stage								
II	Reference				Reference			
III	3.53 (0.47-26.37)	0.22			2.23 (0.80-6.20)	0.12		
Location								
Low	Reference				Reference			
Middle & High	0.98 (0.41-2.34)	0.96			1.40 (0.80-2.46)	0.24		
MAC								
Negative	Reference				Reference			
Positive	2.23 (0.65-7.62)	0.20			2.60 (1.22-5.55)	0.01		
mrT stage								
T2-T3a/b	Reference		Reference		Reference			
T3c/d-T4	9.32 (2.71-32.01)	<0.001	2.95 (0.75-11.58)	0.12	4.71 (2.52-8.79)	<0.001		
mrN stage								
N0	Reference				Reference			
N1-2	4.54 (1.32-15.58)	0.02			3.07 (1.56-6.04)	<0.001		
mrEMVI status								
Negative	Reference				Reference			
Positive	3.90 (1.61-9.44)	<0.01			4.18 (2.39-7.33)	<0.001		
mrTDs status								
Negative	Reference				Reference			
Positive	4.30 (1.78-10.37)	<0.01			6.85 (3.87-12.1)	<0.001		
mrMRF status								
Negative	Reference				Reference			
Positive	12.18 (4.06-36.49)	<0.001			5.37 (3.03-9.54)	<0.001		
mrLLNs status								
Negative	Reference				Reference			
Positive	10.33 (4.27-24.96)	<0.001			3.48 (1.87-6.47)	<0.001		
mrTML score								
0	Reference		Reference	NA	Reference		Reference	
1	7.07 (1.37-36.49)	0.02	4.26 (0.74-24.52)	0.10	5.74 (2.53-13.0)	<0.001	5.74 (2.53-13.0)	<0.01
2	18.34 (3.8-88.48)	<0.001	9.85 (1.76-55.22)	0.01	12.21 (5.4-27.6)	<0.001	12.2 (5.39-27.7)	<0.001
3	74.19 (14.9-370.5)	<0.001	33.99 (6.9-231.1)	<0.001	20.26 (7.8-52.7)	<0.001	20.26 (7.8-52.7)	<0.001

[a]The normal values for CEA level range from 0 to 5 ng/ml.

Abbreviation: EOLARC, early-onset locally advanced rectal cancer; HR, hazard ratio; AHR, adjusted hazard ratio; CI, confidence interval; CEA, carcinoembryonic antigen; cTNM stage, clinical tumor-node-metastasis stage; MAC, mucinous adenocarcinoma; mr, magnetic resonance; EMVI, extramural vascular invasion; TDs, tumor deposits; MRF, mesorectal fascia; LLNs, lateral lymph nodes; mrTML score, the composite score integrating mrTDs, mrMRF, and mrLLNs status.

Supplementary Table 8. Concordance Index Values of Clinical and Imaging Factors for Predicting DFS and OS.

	DFS		OS	
	**C-index**	**95% CI**	**C-index**	**95% CI**
Age	0.510	0.442 - 0.579	0.559	0.452 - 0.666
Gender	0.504	0.447 - 0.561	0.537	0.452 - 0.622
CEA level	0.555	0.497 - 0.613	0.614	0.527 - 0.700
Location	0.524	0.465 - 0.583	0.509	0.423 - 0.596
cTNM stage	0.543	0.515 - 0.570	0.507	0.452 - 0.561
MAC	0.536	0.498 - 0.573	0.574	0.503 - 0.645
mrT stage	0.678	0.629 - 0.726	0.684	0.611 - 0.757
mrN stage	0.610	0.563 - 0.658	0.617	0.555 - 0.680
mrEMVI status	0.651	0.595 - 0.708	0.641	0.557 - 0.725
mrTDs status	0.693	0.637 - 0.748	0.688	0.604 - 0.773
mrMRF status	0.702	0.649 - 0.755	0.711	0.633 - 0.788
mrLLNs status	0.646	0.590 - 0.702	0.583	0.504 - 0.662
mrTML score (EO)	0.787	0.735 - 0.834	0.779	0.711 - 0.848
mrTML score (LO)	0.664	0.624 - 0.704	0.665	0.612 - 0.718
mrTML score (AA)	0.700	0.667 - 0.732	0.692	0.648 - 0.737
mrTEM score (EO)	0.750	0.694 - 0.807	0.777	0.711 - 0.845
mrTEM score (LO)	0.675	0.635 - 0.714	0.678	0.626 - 0.731
mrTEM score (AA)	0.698	0.665 - 0.731	0.703	0.658 - 0.747

Unless otherwise specified, all values refer to patients with early-onset locally advanced rectal cancer.

Abbreviations: DFS, disease-free survival; OS, overall survival; CEA, carcinoembryonic antigen; MAC, mucinous adenocarcinoma; mr, magnetic resonance; EMVI, extramural vascular invasion; TDs, tumor deposits; MRF, mesorectal fascia; LLNs, lateral lymph nodes; mrTML, composite score of mrTDs, mrMRF, and mrLLNs; mrTEM, composite score of mrTDs, mrEMVI, and mrMRF.

Supplementary Table 9. Epidemiologic and Tumor Characteristics in Very Early-Onset vs Early-Onset Rectal Cancer.

Characteristics	Very early-onset (age ≤30 years, N = 35)	Early-onset (30 <age <50 years, N = 352)	*P* value
Age[a] (y)	29 [26-30]	44 [39-47]	<0.001
Gender			
Male	22 (62.9)	227 (64.5)	0.99
Female	13 (37.1)	125 (35.5)	
CEA level[b]			
Normal	22 (62.9)	233 (66.2)	0.83
Abnormal	13 (37.1)	119 (33.8)	
cTNM stage			
II	2 (5.7)	51 (14.5)	0.24
III	33 (94.3)	301 (85.5)	
Location			
Low	17 (48.6)	176 (50.0)	1.00
Middle-High	18 (51.4)	176 (50.0)	
MAC			
Negative	31 (88.6)	327 (92.9)	0.56
Positive	4 (11.4)	25 (7.1)	
mrT stage			
T2	0 (0.0)	2 (0.6)	0.12
T3	26 (74.3)	301 (85.5)	
T4	9 (25.7)	49 (13.9)	
mrN stage			
N0	9 (25.7)	134 (38.1)	0.21
N1-N2	26 (74.3)	218 (61.9)	
mrTDs status			
Negative	23 (65.7)	274 (77.8)	0.16
Positive	12 (34.3)	78 (22.2)	
mrEMVI status			
Negative	21 (60.0)	248 (70.4)	0.28
Positive	14 (40.0)	104 (29.6)	
mrMRF involvement			
Negative	20 (57.1)	244 (69.3)	0.20
Positive	15 (42.9)	108 (30.7)	
mrLLNs status			
Negative	32 (91.4)	298 (84.7)	0.41
Positive	3 (8.6)	54 (15.3)	
pTNM			
0/I	13 (37.1)	160 (45.5)	0.39
II	9 (25.8)	99 (28.1)	
III	13 (37.1)	93 (26.4)	

TRG[c]			
0	8 (22.9)	92 (26.4)	
1	11 (31.4)	92 (26.4)	0.85
2	14 (40.0)	135 (38.6)	
3	2 (5.7)	30 (8.6)	

Unless otherwise indicated, data are No. (%).

[a]Data are medians, with IQRs in parentheses.

[b]The normal values for CEA level range from 0 to 5 ng/ml.

[c]Three cases could not be assessed for TRG due to lack of archival material.

Abbreviation: LARC, locally advanced rectal cancer; CEA, carcinoembryonic antigen; cTNM stage, clinical tumor-node-metastasis stage; MAC, mucinous adenocarcinoma; mr, magnetic resonance; TDs, tumor deposits; EMVI, extramural vascular invasion; MRF, mesorectal fascia; LLNs, lateral lymph nodes; pTNM, pathological TNM stage; TRG, tumor regression

Supplementary Table 10. MRI Acquisition Parameters Across Participating Centers.

Hospital	Scanner	Sequence	TR/TE[a] (ms)	FOV (mm)	Matrix	Slice Thickness	Slice Gap	Flip Angle
**Guangdong provincial People's Hospital**	Philips 3.0T (Ingenia)	T2WI	4300/141.12	100	464×461	4	5.1	90°
		DWI	922.18/65.658	74	108×105	6	7	90°
		CE-T1WI	4/1	121	280×278	4	2	10°
**The Sixth Affiliated Hospital of Sun Yat-sen University**	GE 3.0T (OPTIMA)	T2WI	4300/104	100	288×256	3	6	90°
		DWI	4500/92	100	192×192	3	6	90°
		CE-T1WI	6/3	90	288×256	2	5	12°
**Sun Yat-sen University Cancer Center**	Philips 3.0T (Achieva)	T2WI	2852/90	100	516×510	5	6	90°
		DWI	2374/51	70	132×127	5	5.5	90°
		CE-T1WI	6/3	90	288×285	6	7	10°
	GE 3.0T (DISCOVERY)	T2WI	6480.72/81.692	60	320×224	5	10	90°
		DWI	4000/56.7	100	128×128	6	7	90°
		CE-T1WI	4/2	83	228×256	2	5	11°
	GE 1.5T (SIGNA)	T2WI	6000/92.184	60	320×224	5	10	90°
		DWI	5000/75.6	100	128×128	6	7	90°
		CE-T1WI	4/2	83	228×256	2	5	11°
	SIEMENS 3.0T （Trio Tim）	T2WI	3000/84	75	384×230	5	6	90°
		DWI	4000/70	50	128×64	5	6	90°
		CE-T1WI	5/2	82	228×211	2	4	9°

**Shanxi Cancer Hospital (train cohort)**	Philips 3.0T (Achieva)	T2WI	3000/80	100	300×223	3	3.3	90°
		DWI	2750/53	80	124×187	5	5.5	90°
		CE-T1WI	623.638/20	115	408×367	6	7.5	90°
**Yunnan Cancer Hospital**	SIEMENS 1.5T （Avanto）	T2WI axial	3200/100	100	288×320	4	4	90°
		DWI	4900/84	80	220×220	4	6	90°
		CE-T1WI	5/2	75	320×163	6	3	10°
	Philips 3.0T (Achieva)	T2WI	2100/100	100	400×284	4	4	90°
		DWI	3070/62	100	108×129	3	6	90°
		CE-T1WI	3/1	90	269×250	5	5	10°

Abbreviations: T2WI, T2-weighted images; DWI, diffusion weighted imaging; CE-T1WI, contrast enhanced-T1 weighted imaging; TR, Repetition Time; TE, Echo Time; FOV, Field of View.

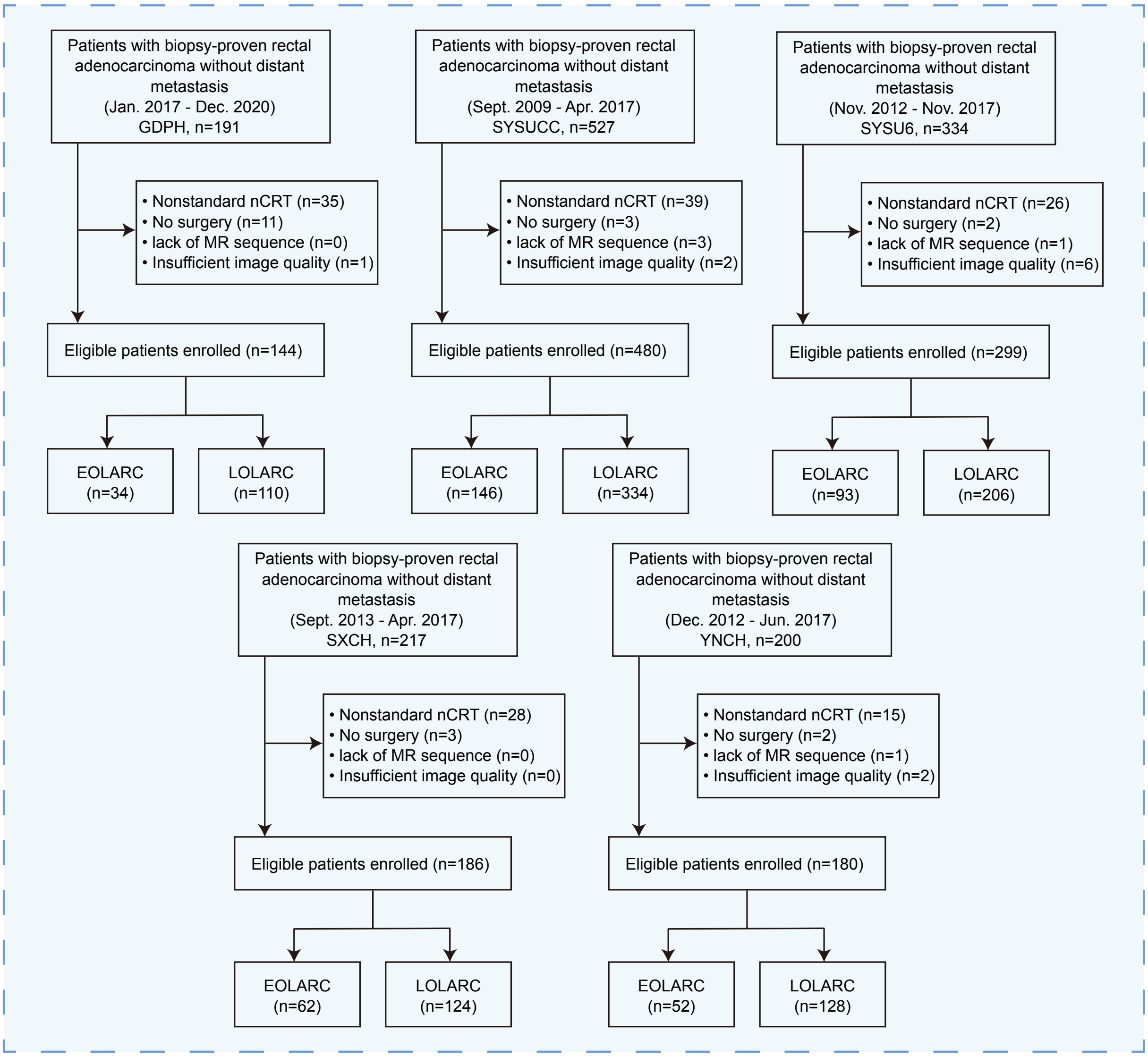

Supplementary Figure 1. Flowchart of the Patient Recruitment Process. Abbreviation: GDPH, Guangdong Provincial People's Hospital; SYSUCC, Sun Yat-sen University Cancer Center; SYSU6, the Sixth Affiliated Hospital of Sun Yat-sen University; SXCH, Shanxi Cancer Hospital; YNCH, Yunnan Cancer Hospital.

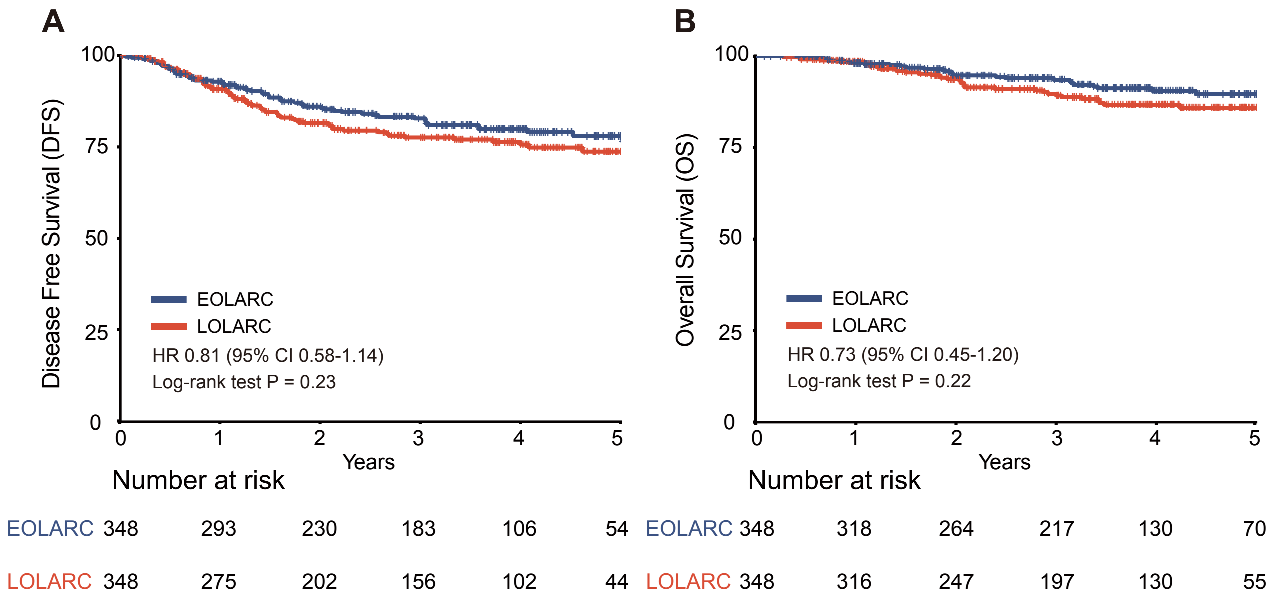

Supplementary Figure 2. Kaplan-Meier Survival Analysis in the Propensity Score-matched Cohort. Comparison of (A) disease-free survival (DFS) and (B) overall survival (OS) between early-onset (EOLARC; age <50 years) and late-onset (LOLARC; age ≥50 years) locally advanced rectal cancer patients after propensity score matching. P-values were calculated using the log-rank test. Hazard ratios (HR) and 95% confidence intervals (CI) are displayed.

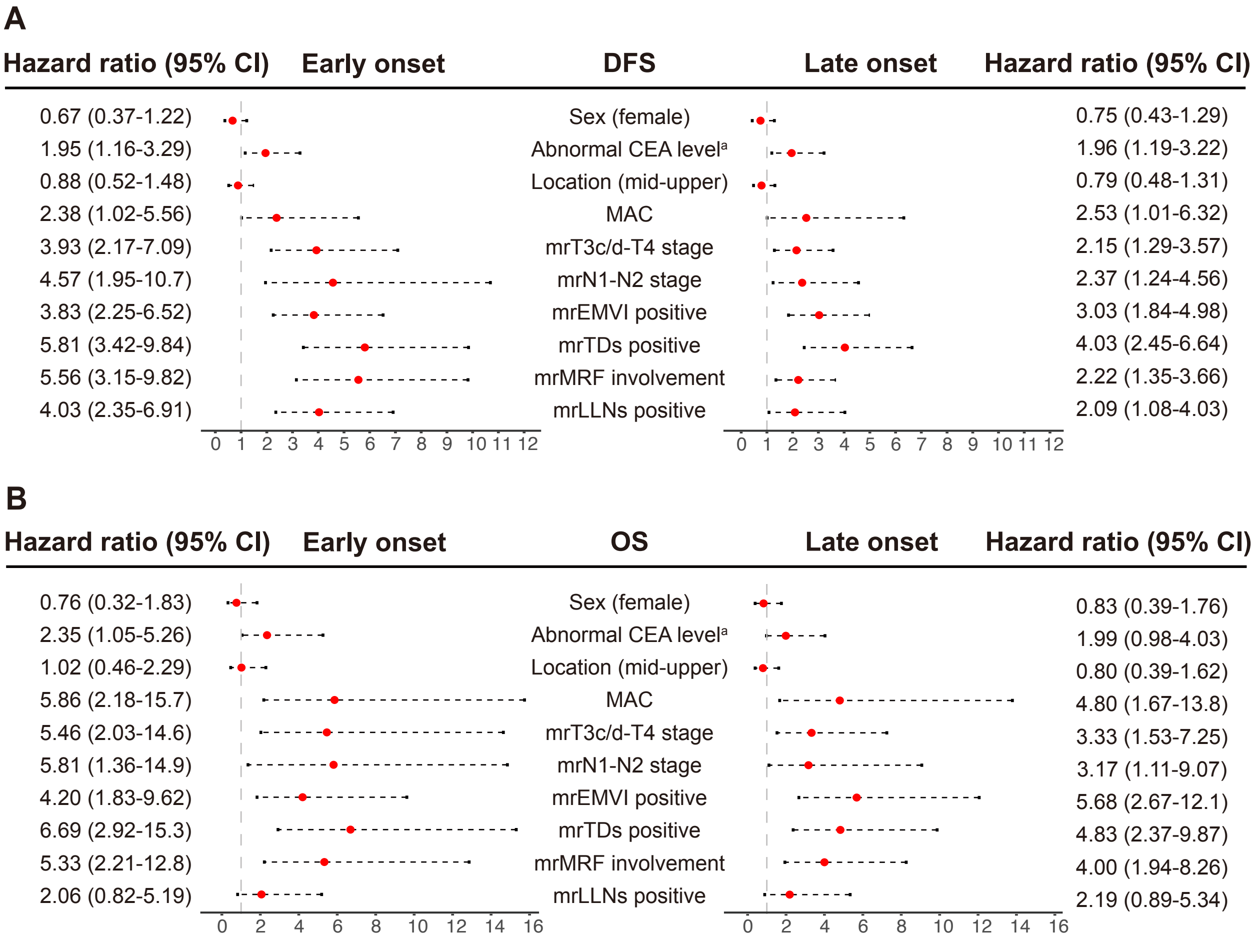

Supplementary Figure 3. Comparison of Prognostic Factors Between Early-Onset and Late-Onset Locally Advanced Rectal Cancer in Stage III After Propensity Score Matching. (A, B) Forest plots of univariate Cox regression analyses showing hazard ratios (HRs) and 95% confidence intervals (CIs) for (A) disease-free survival (DFS) and (B) overall survival (OS). The analyses are stratified by age group: the left panel represents early-onset locally advanced rectal cancer (EOLARC; age <50 years), and the right panel represents late-onset locally advanced rectal cancer (LOLARC; age ≥50 years). Dashed vertical lines indicate the reference value (HR = 1.0). Abbreviations: CI, confidence interval; CEA, carcinoembryonic antigen; cTNM, clinical tumor-node-metastasis; MAC, mucinous adenocarcinoma; mr, magnetic resonance; TDs, tumor deposits; EMVI, extramural vascular invasion; CRM, circumferential resection margin; LLNs, lateral lymph nodes.

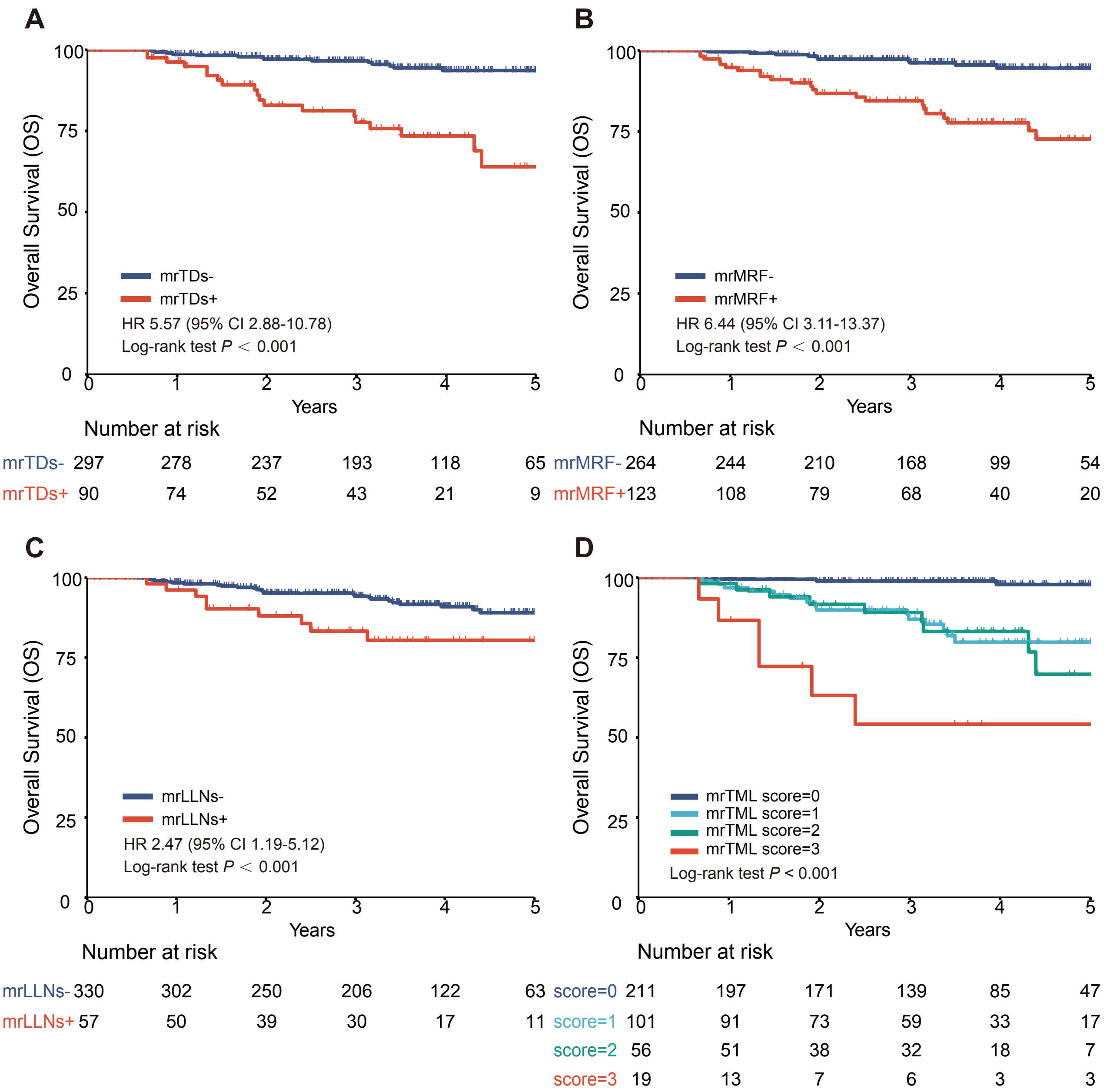

Supplementary Figure 4. Prognostic Impact of MRI Biomarkers in EOLARC. Kaplan-Meier curves for Overall Survival (OS) stratified by (A) MRI-detected tumor deposits (mrTDs), (B) mesorectal fascia involvement (mrMRF), (C) lateral lymph node metastasis (mrLLNs) and (D) the mrTML score (integrated by mrTDs, mrMRF, and mrLLNs status), respectively. Abbreviations: EOLARC, early-onset locally advanced rectal cancer; HR, hazard ratio.

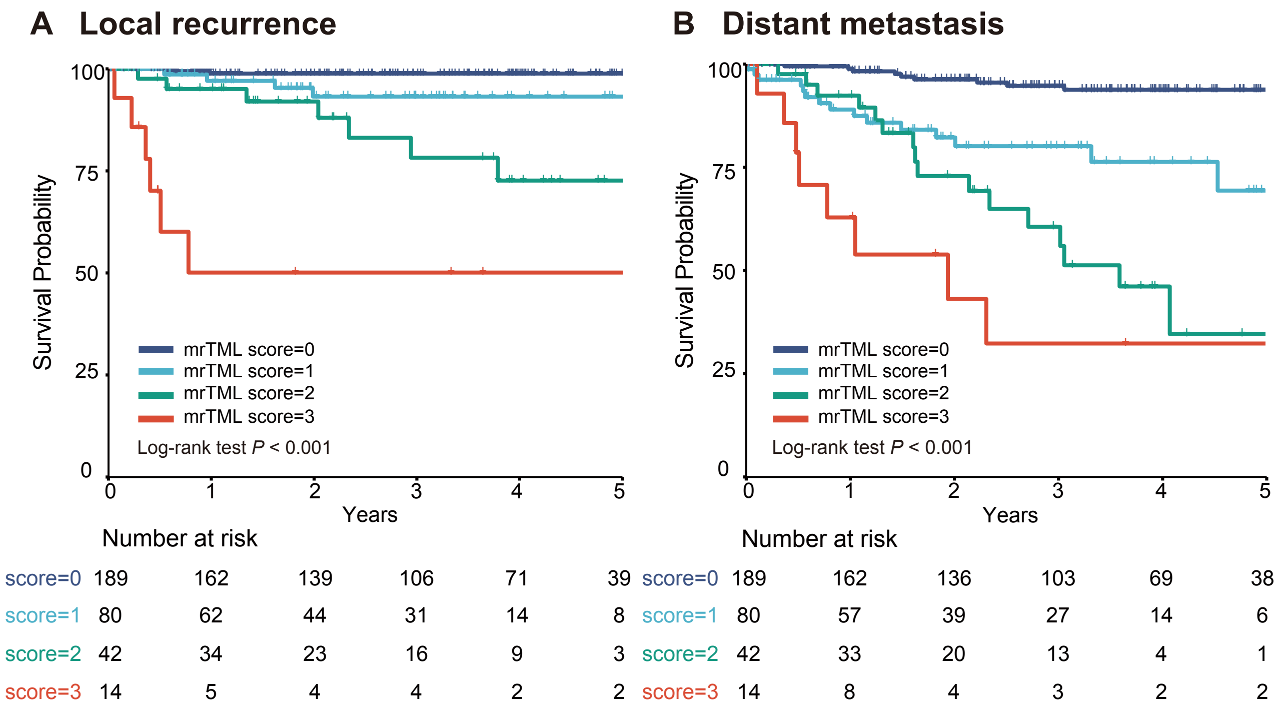

Supplementary Figure 5. Prognostic Impact of mrTML Score in EOLARC. Kaplan-Meier survival curves for (A) Local recurrence (LR) and (B) Distant metastasis (DM) stratified by mrTML score. The mrTML score integrates mrTDs, mrMRF, and mrLLNs status. Abbreviations: EOLARC, early-onset locally advanced rectal cancer.

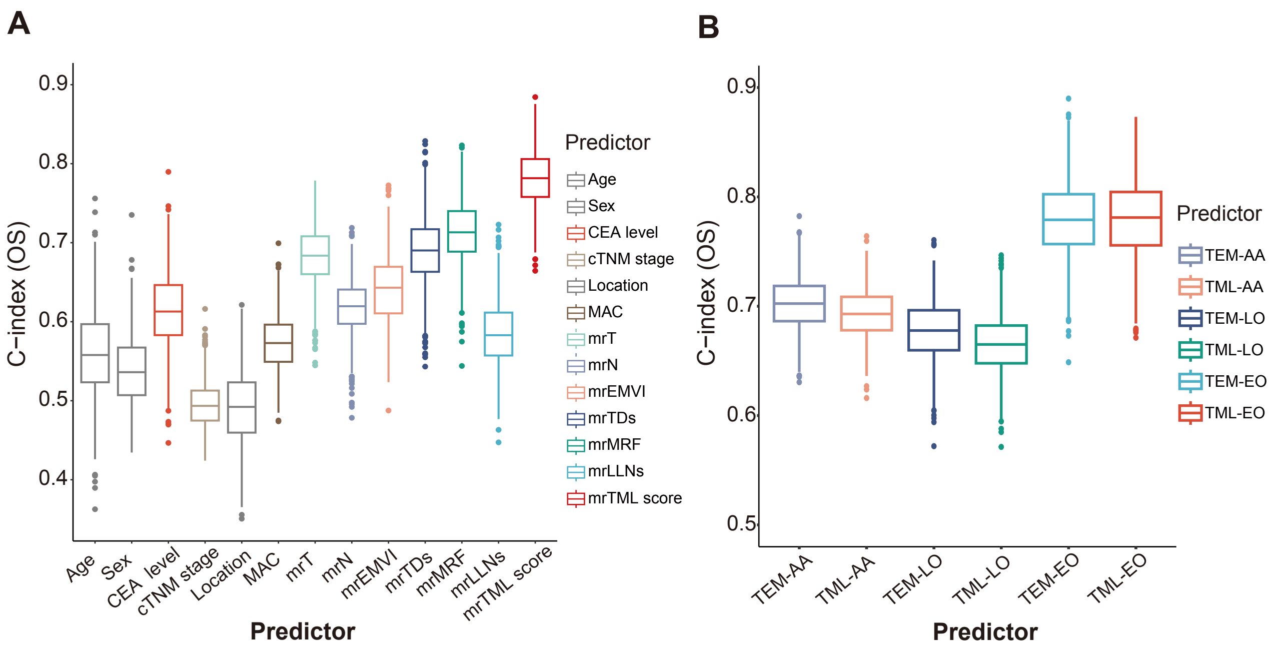

Supplementary Figure 6. Prognostic Performance for Predicting Overall Survival (OS) in EOLARC. (A) Box plots illustrating the distribution of Harrell's concordance index (C-index) for predicting OS across 1,000 bootstrap resamples, comparing the mrTML score with individual clinical and imaging predictors. (B) Box plots comparing C-index values between the mrTML and mrTEM scores (the latter comprising mrTDs, mrEMVI, and mrMRF status) across different age-stratified cohorts: all-age (AA), early-onset (EO; age <50 years), and late-onset (LO; age ≥50 years). Each box represents the median (center line), interquartile range (box edges), and full range (whiskers). Abbreviations: EOLARC, early-onset locally advanced rectal cancer; CEA, carcinoembryonic antigen; cTNM, clinical tumor-node-metastasis stage; MAC, mucinous adenocarcinoma; mr, magnetic resonance; TDs, tumor deposits; EMVI, extramural vascular invasion; MRF, mesorectal fascia; LLNs, lateral lymph nodes; C-index, concordance index.

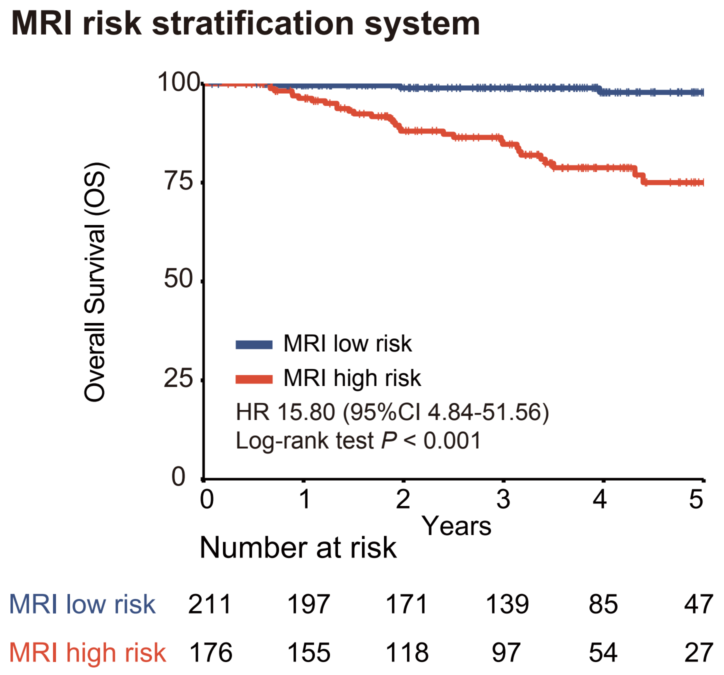

Supplementary Figure 7. Kaplan-Meier Curves of Overall Survival (OS) Stratified by MRI-Based Risk Stratification in Early-Onset Locally Advanced Rectal Cancer.

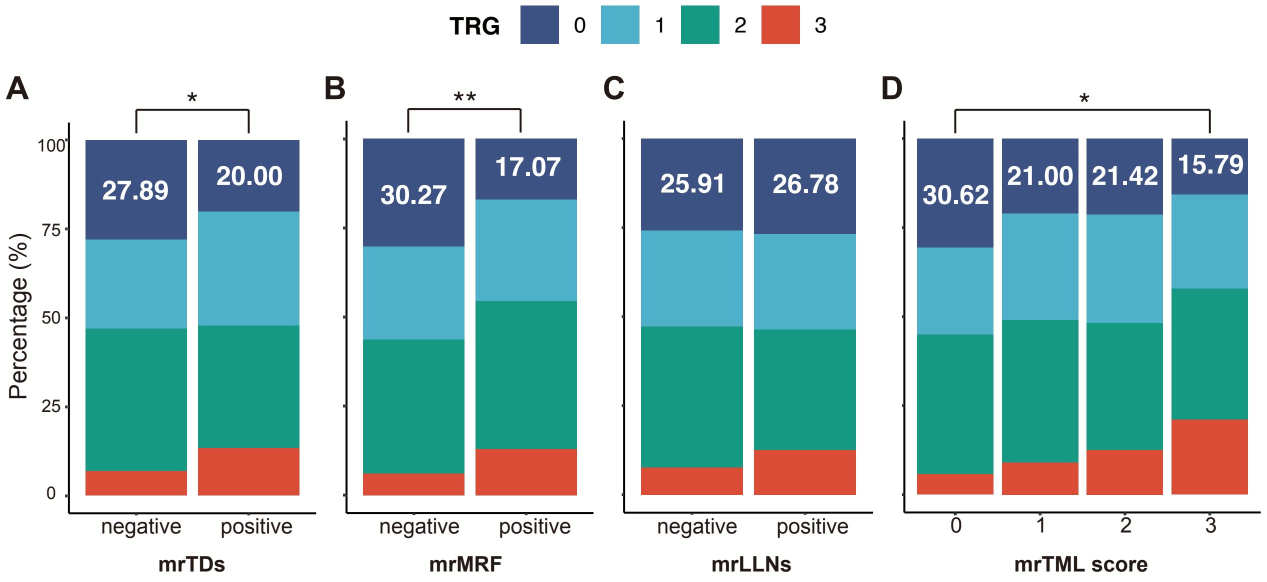

Supplementary Figure 8. Association Between MRI-Based Risk Stratification and Treatment Response in EOLARC. Stacked bar chart showing tumor regression grade in EOLARC patients. Efficacy analysis of neoadjuvant therapy according to (A) mrTDs status, (B) mrMRF status, (C) mrLLNs, and (D) mrTML score. Percentages indicate response distributions within the matched cohorts. Abbreviations: EOLARC, early-onset locally advanced rectal cancer (age <50 years); mr, magnetic resonance; TDs, tumor deposits; MRF, mesorectal fascia; LLNs, lateral lymph nodes; TRG, tumor regression grade. * P value < 0.01, ** P value < 0.05.

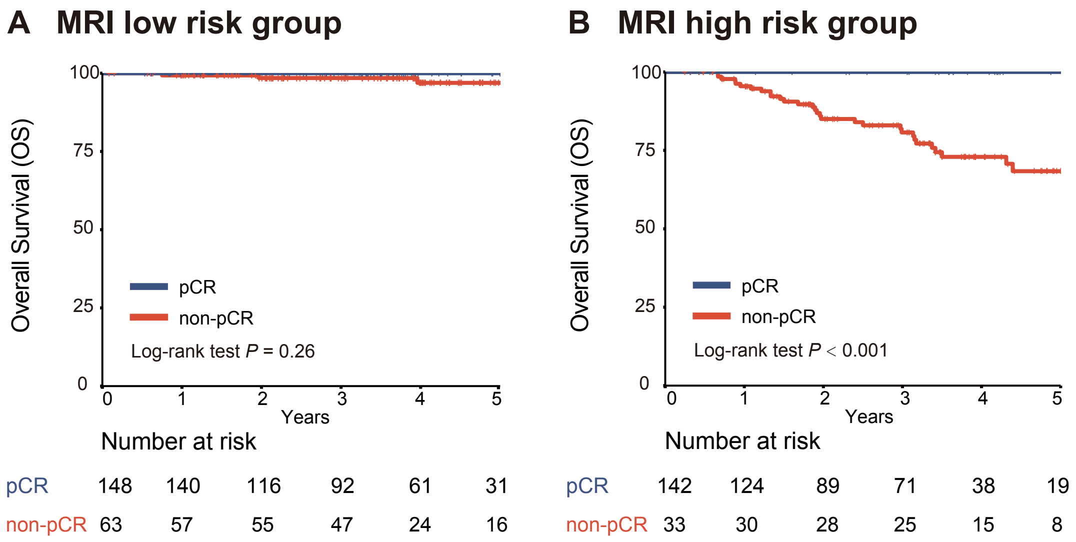

Supplementary Figure 9. Prognostic Stratification by Pathological Response Status in MRI-Based Risk Subgroups. Kaplan-Meier survival curves for overall survival (OS) in the (A) MRI low-risk group and (B) MRI high-risk group, comparing patients with pathological complete response (pCR) and non-pCR. Abbreviations: pCR, pathological complete response (ypT0N0).

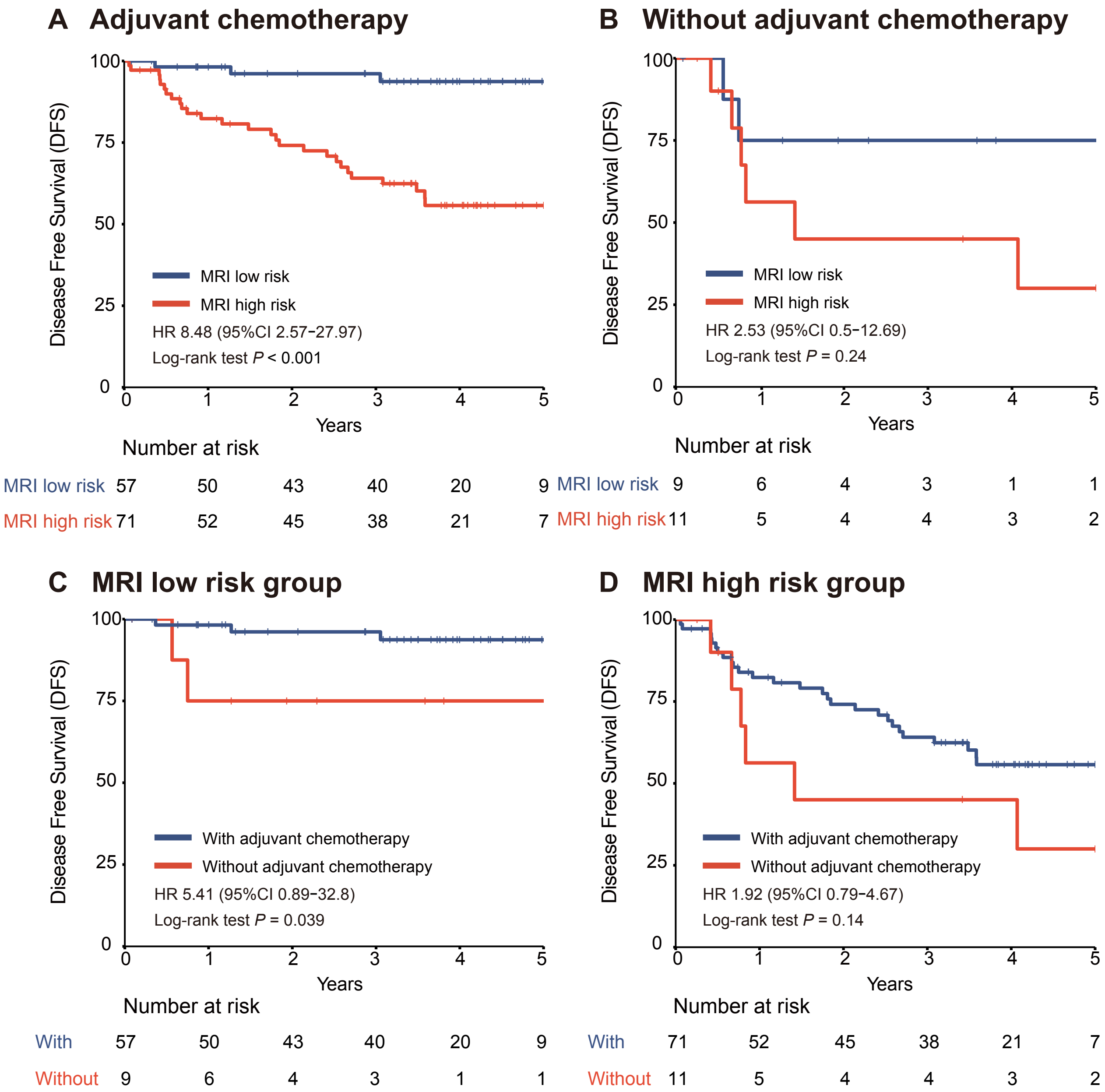

Supplementary Figure 10. Impact of MRI Risk Stratification and Adjuvant Chemotherapy on Disease-Free Survival (DFS) in Early-Onset Locally Advanced Rectal Cancer. (A, B) Kaplan-Meier curves comparing MRI low-risk versus high-risk patients (A) with adjuvant chemotherapy and (B) without adjuvant chemotherapy. (C, D) Kaplan-Meier curves comparing patients with versus without adjuvant chemotherapy in the (C) MRI low-risk group and (D) MRI high-risk group.

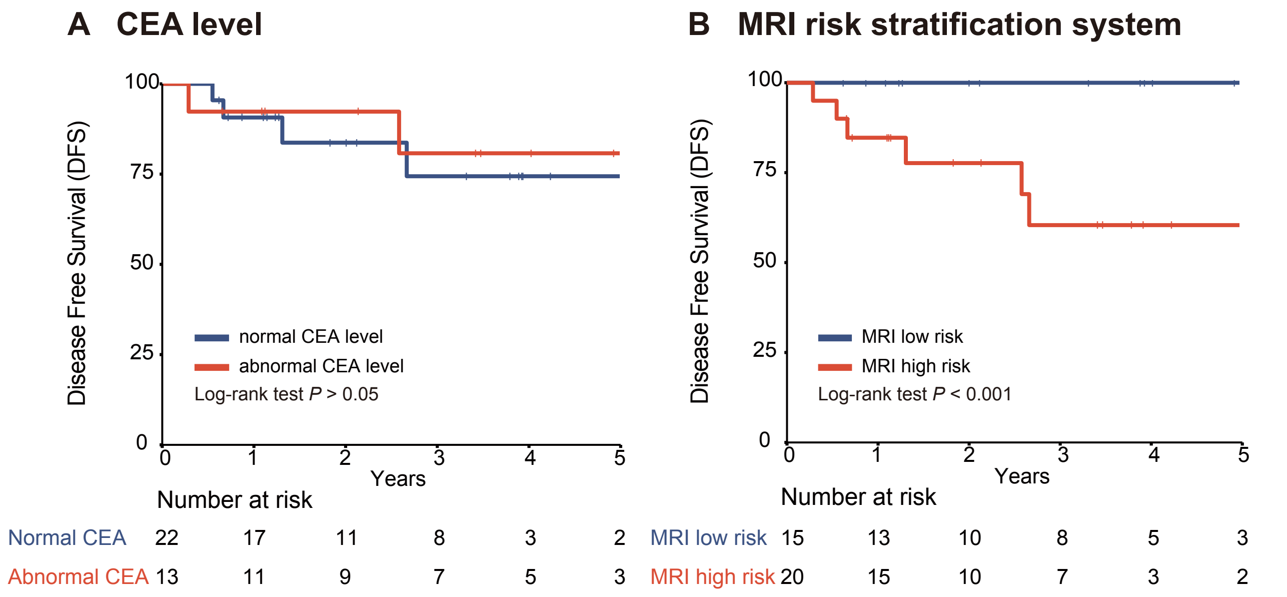

Supplementary Figure 11. Prognostic Impact of CEA Level and MRI-Based Risk Stratification in Very Early-Onset (Age ≤30 Years) Locally Advanced Rectal Cancer. Kaplan-Meier analysis of disease-free survival (DFS) stratified by (A) CEA level and (B) MRI-based risk stratification. Abbreviations: CEA, carcinoembryonic antigen (normal <5 ng/mL).
